# Quality assurance in orthognathic surgery using Brons-Mulié's soft tissue analysis, Nakamura's asymmetry index and a simple enface analysis

**DOI:** 10.3389/froh.2026.1748425

**Published:** 2026-03-26

**Authors:** Sebastian Böttger, Sina Herrmann, Eva May Schraml, Nina Danevitch, Jörn Pons-Kühnemann, Rob Mulié, Hans-Peter Howaldt, Philipp Streckbein

**Affiliations:** 1 Department of Oral and Maxillofacial Surgery, Justus-Liebig-University Giessen, University Hospital Giessen, Giessen, Germany; 2 Institute of Medical Informatics, Justus-Liebig-University Giessen, Giessen, Germany; 3 Orthodontist, Orfeokliniek, Zoetermeer, Holland

**Keywords:** Brons-Mulié analysis, enface analysis, facial harmony, facial photography, nakamura asymmetry index, orthognathic surgery

## Abstract

The aim of orthognathic surgery should not only focus on correcting malocclusion alone, but also on achieving adequate facial aesthetics with a harmonious facial profile. However, it is often difficult to measure success of orthognathic surgery objectively due to the large number of parameters to be considered.

**Methods:**

For objective assessment of success in orthognathic surgery, Brons-Mulié profile analysis, Nakamura's asymmetry index and a simple enface analysis were performed pre- and postoperatively. Comparative variance-based analysis of the different dimensions of the three methods were performed in order to analyze the variability of the individual parameters around their optimal value. *F*-test and Fligner-Killeen-Test were used to determine differences between pre- and post-operative variances.

**Results:**

A total of 128 patients who underwent bimaxillary surgery between 2014 and 2019 were included in the study. All parameters showed a constant or reduced variance around their optimal value after orthognathic surgery. A significant reduction of the variance as an expression of surgical success (*p* < 0.05) was found for the following parameters: Mandibulofacial height and lower lip inclination obtained from Brons-Mulié analysis, position of the nostrils, lip angles and jaw angles using Nakamura's Asymmetry Index and for chin position, occlusal plane inclination and midline position measured in the enface analysis.

**Conclusions:**

The methodology used allows subjective aesthetic appearance to be visualized in an objective procedure. It can therefore be used as a valuable tool for preoperative surgical planning and for postoperative quality assurance in orthognathic surgery.

## 1 Introduction

Orthognathic surgery is one of the most commonly performed surgical procedures in oral and maxillofacial surgery. The aim is not only to achieve a stable and neutral occlusion, but also to create a good aesthetic appearance with a harmonious facial profile. Comprehensive preoperative planning is usually carried out with preparation of occlusal and cephalometric analyses and subsequent surgical simulation using an articulator or virtual 3D-planning software (1). As described by Weiss et al., the surgical team should have a strong foundation in facial analysis and firm understanding of the maxillofacial skeleton in order to achieve surgical success (2). According to Steenen et al., patients regard the achievement of a favorable aesthetic appearance to be of equal importance as the establishment of good oral function (3). Therefore, the preoperative cephalometric analyses are particularly important, as they are able to indicate the direction of therapeutic corrections from abnormal to normal. Until today, 2D analyses based on the lateral cephalogram and standardized photographs of the face and skull from different perspectives are frequently used for this purpose (4–7). In the frontal view, often bilateral pairs of points are defined and their symmetry is assessed using a baseline (5), while in the profile view from the lateral, the shape of the profile line is usually examined (8–11). This approach and also the use of the golden ratio to compare different facial proportions partly originate from the work of Leonardo da Vinci (4).

With the introduction of modern Cone Beam CT imaging and the possibility of 3D surface scans, very sophisticated three-dimensional visualizations and analysis methods are increasingly available, but these have not yet been able to replace the previous standards of 2D analyses on a broad scale (4, 7). In fact, even two-dimensional layers are often extracted from three-dimensional imaging in order to analyze them with the proven 2D methods (4, 12). Many considerations and analyses of facial beauty are also based on two-dimensional concepts (5, 8, 13). Therefore, even in a modern three-dimensional world, 2D analyses are still an indispensable standard of orthodontic and orthognathic diagnostics (4, 7).

The Brons-Mulié method, Nakamura's asymmetry index and a simple enface analysis are proven and published 2D analysis methods based on standardized clinical photographs (5, 8, 11, 14). These methods can be applied as often as required without the use of any x-rays on the basis of simple technology. Moreover, they have been developed to objectively describe the aesthetic appearance of the face (5, 8, 13).

This study aims to evaluate whether a straightforward, free-to-use digital analysis tool, based on established 2D methods, can be used for both planning orthognathic surgery and assessing postoperative outcomes, and whether it can effectively distinguish between favorable and less favorable results. In this context, the easily applicable 2D methods are intended to complement, rather than replace, modern 3D planning procedures. Since the data collection for this study took place during the transition period from the old articulator-based, analog planning to software-based, digital planning, the methods should also be used to investigate whether differences between analogously and digitally planned surgeries can be determined.

## 2 Materials and methods

### 2.1 Preparation of analyses before and after orthognathic surgery

**Figure 1:**
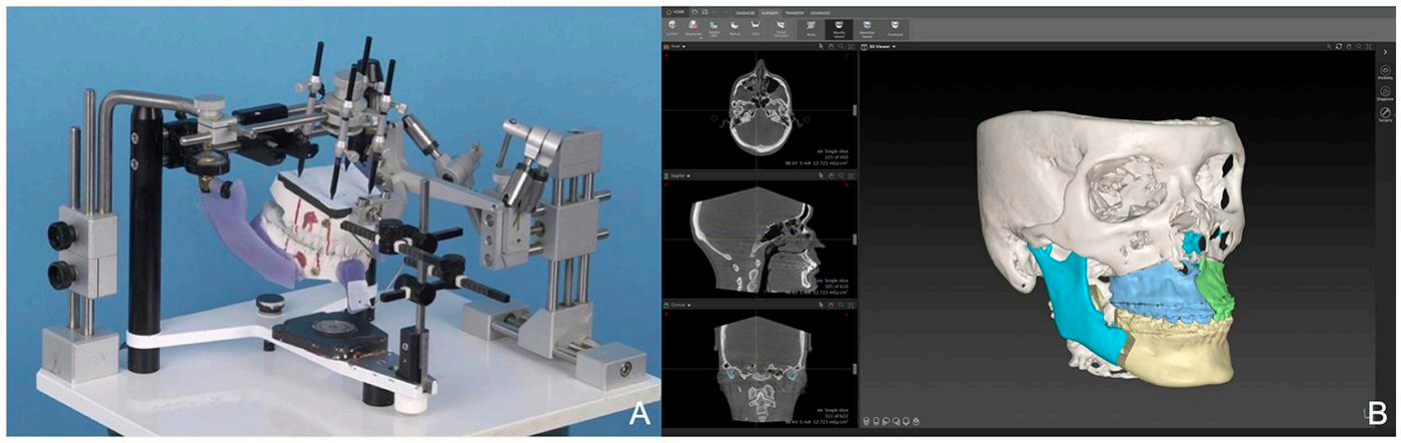
Planning of orthognathic surgery. **(A)** Analog planning with the 3D-OSS articulator (Girrbach-Dental, Pforzheim, Germany); **(B)** Digital planning with the IPS CaseDesigner (KLS Martin, Tuttlingen, Germany).

For each orthognathic intervention, a soft tissue profile analysis according to Brons and Mulié was performed (8) as well as an asymmetry index according to Nakamura using four landmarks (5, 14) and a simple enface analysis (15) before and after surgery. The analyses were carried out on the basis of frontal and profile photographs of the face taken from a distance of approximately 150 cm with a digital SLR camera (Canon EOS 6D; ISO-Sensitivity: 200; Aperture: f/18; shutter speed to 1/125 s; external flash units) against a blue background in accordance with published standards (6, 16). The photographs were acquired under strictly standardized conditions in a professional photography studio to minimize measurement variability arising from the imaging technique. Object-to-lens distance, exposure settings, and patient positioning were kept constant across all sessions to ensure comparability of the images. The entire acquisition procedure, along with the estimated standard error due to depth distortion, are described in detail in Supplementary Appendix C. The preoperative analyses were carried out approximately 6 weeks prior to surgery and the postoperative analyses approximately 6 months after surgery. The surgical interventions were planned either analogously with the 3D-OSS articulator (Girrbach-Dental, Pforzheim, Germany) according to Krenkl and Litzl (17) or digitally with the IPS CaseDesigner (KLS Martin, Tuttlingen, Germany) (18), where all the preoperative 2D analyses were used as planning aids. Using analog planning, surgical wafers were produced in the articulator by a dental technician, while digital planning was carried out using CAD-CAM-technology with a 3D-printing process (Figure 1). Both, the 3D-OSS articulator and the IPS CaseDesigner enable translational and rotational movements in all three dimensions of space (17, 18). The aim of the planning was to achieve not only a neutral occlusion but also the most aesthetically pleasing result possible through the subsequent surgical procedure.

### 2.2 Profile analysis according to Brons and Mulié

**Figure 2:**
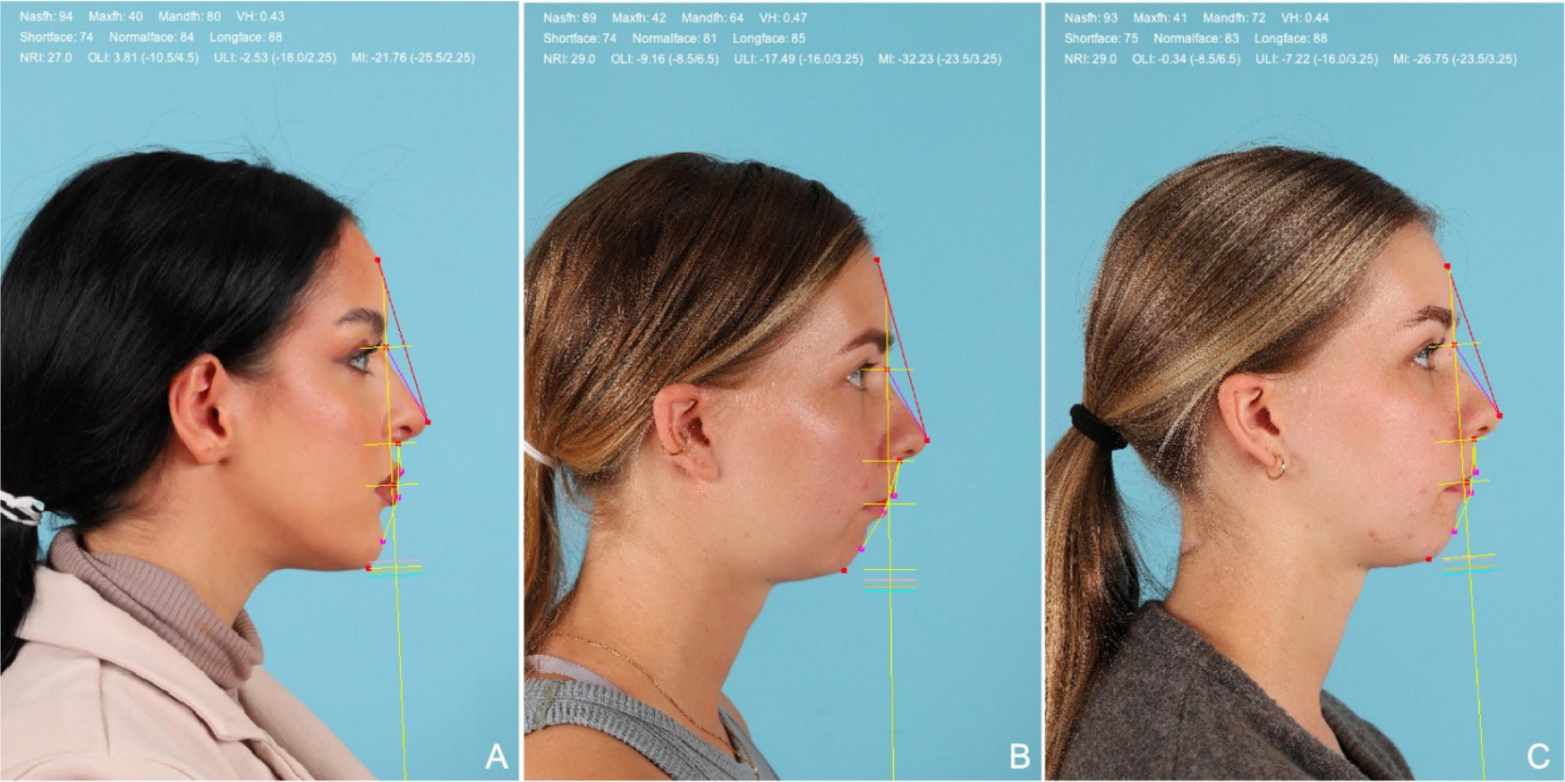
Profile analysis according to Brons and Mulié (11). Left photo **(A)** shows a profile with satisfactory facial harmony in vertical and sagittal dimensions. Right photos show the preoperative **(B)** and the postoperative **(C)** profile of a patient with Class II malocclusion. This profile could be significantly improved but was still not perfect after surgery, because the chin was still too short in both, the vertical and the sagittal dimension.

The Brons-Mulié analysis was carried out as described by Brons on the basis of 2D profile photographs before and after surgery (8, 11). To simplify the preparation of the analyses, a small JAVA program was developed to digitally create the required points, distances and angles with just a few clicks (Figure 2 and Supplementary Appendix E). Depending on the naso-frontal line, an individual vertical analysis line (VAL) is created, which is used to assess the facial profile in the vertical and sagittal dimensions (8). In the vertical dimension, the mandibulofacial height (MandFH) is determined along this line as the distance between the lip commissure and the point mentum (gnathion). The MandFH has to be compared with the individual optimum value (normal face) and a lower and an upper threshold. Thus, a too long lower face is classified as a long face constellation and a too short face is rated as a short face constellation. In the sagittal dimension, the upper lip inclination, the lower lip inclination and the mandibular inclination are determined as angles to VAL and compared with the respective individual normal range. A profile that is too retrogenic is therefore classified as a dorsal characteristic and a profile that is too progenic is rated as a ventral characteristic. According to Brons and Mulié, facial harmony is achieved when the profile line runs within the normal value corridors (8, 10, 11).

### 2.3 Facial analysis applying the Nakamura asymmetry index

**Figure 3:**
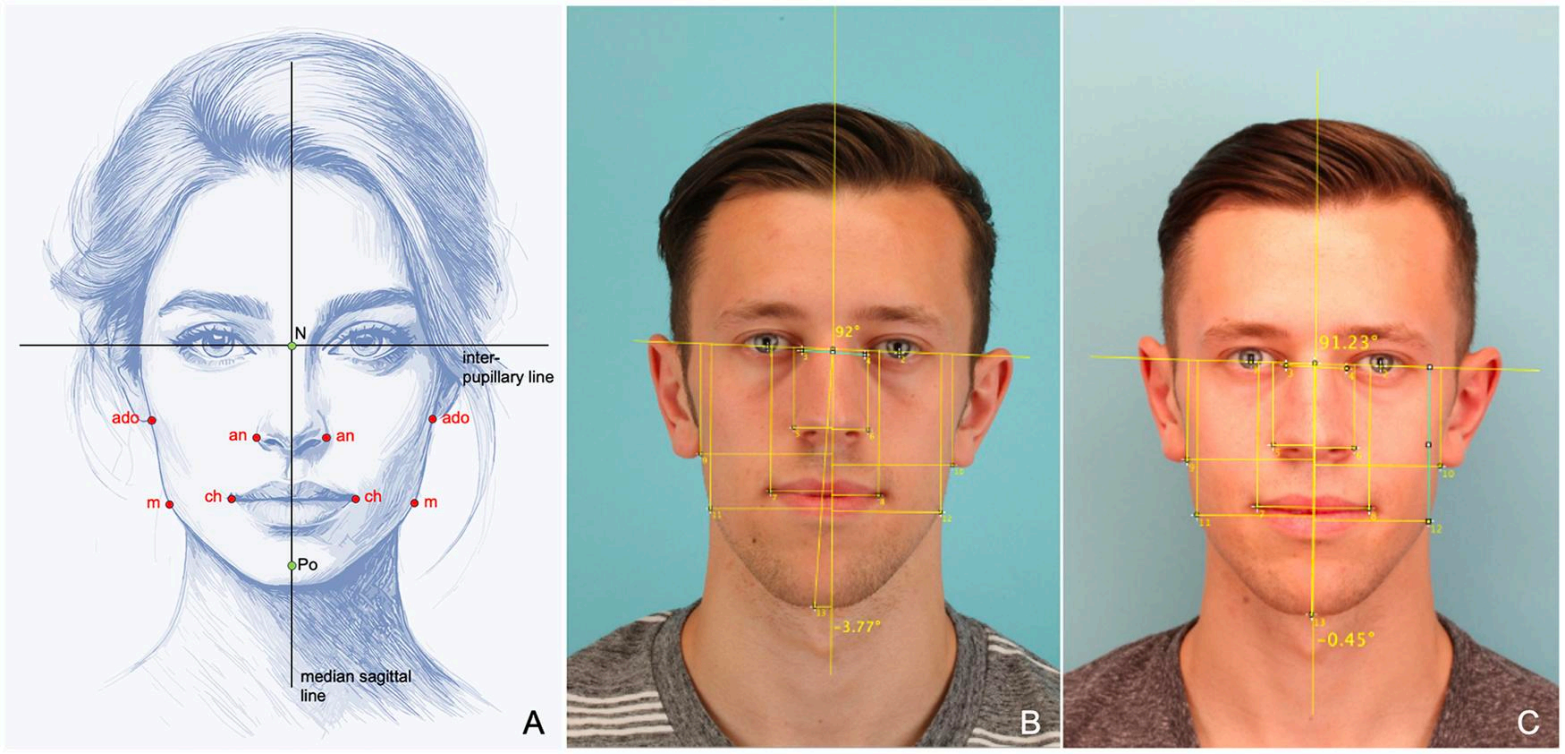
Calculation of nakamura asymmetry indices: for the horizontal assessment, the nakamura asymmetry indices are calculated from the distances of the red-coloured points to the median sagittal line and for the vertical evaluation to the interpupillary line (**A**) - the lower the resulting AI values, the better the symmetry of the face. Left photos show a preoperative (**B**) and postoperative (**C**) analysis using ImageJ program with a strong improvement of symmetry by surgery.

The creation of the asymmetry index (AI) is based on studies by Nakamura on the frequency of complaints in the head and neck area in the context of asymmetries of the face (14). In their review, Berlin et al. describe that an AI can be determined for each pair of points in an enface photograph measuring the distances to a reference line in both, the horizontal and vertical dimension (5). In the horizontal dimension, the median sagittal line and in the vertical dimension the interpupillary line are used as reference (5). The distances between the points and the references on both sides are measured (*dr*, *dl*) and related to each other using the AI-formula (14) (Figure 3):

$$AI=\left|\frac{dr-dl}{dr+dl}\right|$$

In the presence of ideal symmetry, an AI-value of 0 is obtained for a pair of points. The greater the AI-value, the greater is the asymmetry of the pair of points (5, 14). As described by Berlin et al., it is possible to determine an average AI for several pairs of points (5). In this study the points **an** (lateral margin of the nasal wing, outermost point), **ch** (corner of the mouth), **m** (outermost point of the angle of the mandible) and **ado** (lower corner of the ear) were used separately according to Berlin et al. (5) (Figure 3). All AI determinations were performed using the ImageJ program and with a special JScript developed for this purpose (19). AI was calculated in the horizontal (h) and vertical dimension (v) as AI Nasal wing for the point **an**, AI Lip angle for the point **ch**, AI Jaw angle for the point **m** and AI Earlobe for the point **ado**.

### 2.4 Enface analysis

**Figure 4:**
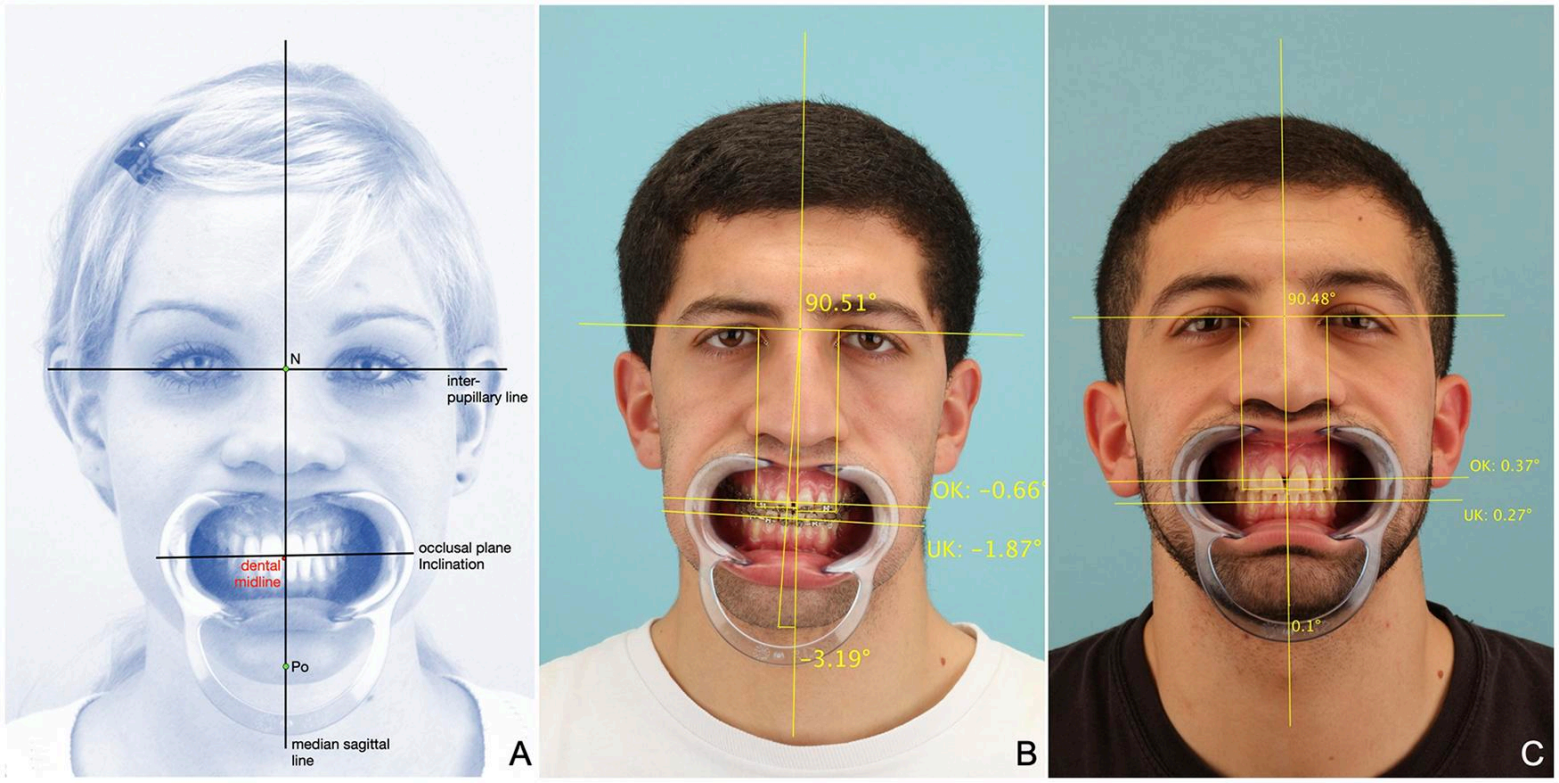
Enface analysis. Three essential parameters of the analysis are the deviation of the chin, the midline shift of the maxilla and the inclination of the occlusal plane in relation to the interpupillary line. Here, the deviation of the chin is specified as the angle between the median sagittal line and the line connecting the points N (nasion) and Po (pogonion) (5).

Planning orthognathic surgery, the correct alignment of dental midline and chin to the median-sagittal line is an important prerequisite for achieving symmetry and favorable facial aesthetics (15, 20). Furthermore, the frontal occlusal plane should be as parallel as possible to the interpupillary line (15). These parameters can be easily determined in a simple analysis of an enface photograph if the perioral soft tissues are retracted with a cheek retractor (Figure 4). As described by Ko et al., the deviation of the chin can be well described as angle to the mid-sagittal line above the nasion point (chin angle) (15), while the frontal inclination angle of the occlusal plane can be well determined in comparison to the interpupillary line (15). In the best case both angles should amount to zero degrees and both are independent of the magnification scale of the photograph. The deviation of the dental midline from the mid-sagittal line can be specified as distance, whereby magnification scales have to be adjusted if different photographs are compared. An invariable line, such as the inner intercanthal distance, can be used to adjust the scales. In the present study, ImageJ software with a separate Jscript was used to determine the chin angle, the inclination of the frontal occlusal plane and the deviation of the dental midline using an enface photograph with an inserted cheek retractor.

### 2.5 Transformation into one evaluation method

A total of 15 continuous parameters were selected from the three analysis methods and transferred to a uniform quality assurance instrument. With the exception of parameters originating from the Brons-Mulié Analysis, all parameters were already centered around an optimal value of zero by definition. Mandibulofacial height was converted into a zero-centered variable by expressing the relative deviation from the optimal value (“normal face”), as detailed in Table 1. The sagittal parameters (upper lip inclination, lower lip inclination, and mandibular inclination) were centered around zero by calculating the difference between the measured value and the mean of the respective normal range. To allow joint visualization across heterogeneous measures, a linear rescaling was performed according to the formula

$$\mathrm{Paramete}r_{i}=x_{i}\times F_{i}$$

where *x_i_* denotes the original signed measurement (pixel or angular degree) and *F_i_* a predefined, parameter-specific transitional factor (Table 1). These factors were selected solely to harmonize numerical ranges while preserving sign and relative differences.

**Table 1:** Evaluation method consisting of a total of 15 parameters taken from Brons-Mulié analysis, Nakamura asymmetry Index and Enface analysis.

Parameter	Original unit	Description	Transitional factor
Brons-Mulié analysis:			
Mandibulofacial Height	Pixel	(Mandibulofacial Height/Normal Face)—1	1
Upper lip inclination	Angular degree	Deviation from mean value of normal range	0.01
Lower lip inclination	Angular degree	Deviation from mean value of normal range	0.01
Mandibula inclination	Angular degree	Deviation from mean value of normal range	0.01
Nakamura index (horizontal):			
AI Nasal wing (h)	Pixel	Deviation from Zero	1
AI Lip angle (h)	Pixel	Deviation from Zero	1
AI Jaw Angle (h)	Pixel	Deviation from Zero	1
AI Earlobe (h)	Pixel	Deviation from Zero	1
Nakamura index (vertical):			
AI Nasal wing (v)	Pixel	Deviation from Zero	1
AI Lip angle (v)	Pixel	Deviation from Zero	1
AI Jaw Angle (v)	Pixel	Deviation from Zero	1
AI Earlobe (v)	Pixel	Deviation from Zero	1
Enface analysis:			
Chin deviation	Angular degree	Deviation from Zero	0.1
Occlusal-plane inclination	Angular degree	Deviation from Zero	0.1
Midline shift	Pixel	Deviation from Zero	0.01

Parameter-specific transitional factors were used to allow the parameters to be represented on the same scale.

Accordingly, mandibulofacial height values exceeding the optimal value “normal face” resulted in positive values, whereas lower values yielded negative results. The sagittal parameters assumed increasingly positive values with greater anterior deviation from the optimum and increasingly negative values with greater posterior deviation. In general, larger deviations from the optimal values corresponded to greater distances from zero. The asymmetry index *a priori* has the optimum value of zero in the case of perfect symmetry between two points and deviations only occur in a positive direction due to the absolute value function of the AI-formula. The inclination angle of the occlusal plane to the interpupillary line, the chin angle and the deviation of the midline also have a zero value as optimum, but similarly to the values of the Brons-Mulie analysis, they deviate in both directions. Table 1 lists the transformations of all 15 parameters.

### 2.6 Statistical analysis

This study is a retrospective, descriptive, and exploratory investigation, with the reduction of parameter variance as the primary endpoint. All patients, who underwent orthognathic surgery between September 2014 and December 2019 and for whom complete photo documentation was available pre- and postoperatively, were included. Statistical analysis was carried out with Microsoft Excel (Redmond, WA, USA) and the statistical software R-4.0.4 (R Core Team, 2021, Vienna, Austria). Differences between the analog and digitally planned patients were tested for significance using Fisher's exact test and Mann–Whitney *U*-test. Pre- and postoperative presentation of deviations from the optimal value of zero were presented using density plots and boxplots. Differences between pre- and postoperative deviations were examined using the parametric *F*-test and the non-parametric FK-test for variances. The study hypothesis was that the variance of deviations from the optimal value of zero would be greater preoperatively than postoperatively, as orthognathic surgery aims to harmonize and symmetrize the face (8, 15). Variance reduction was chosen as the primary endpoint for two main reasons. First, patients present with a broad and heterogeneous spectrum of dental and skeletal malformations, and the corresponding orthodontic and surgical interventions can vary substantially, making pairwise comparisons less meaningful. Second, for several facial parameters, many patients already have optimal or near-optimal values preoperatively. In these cases, the clinical goal is to preserve the preoperative state, and a paired analysis of raw changes would fail to capture this intended effect. The level of statistical significance was set at 0.05.

False discovery rate was controlled using the Benjamini-Hochberg procedure. The final graphical representation of changes from pre- to postoperative was carried out using straight lines, whereby an improvement of a parameter was represented by an approximation of the straight line to zero, while a deterioration was represented by an extension of the distance from zero. Inter- and intra-observer reliability of the measurements was assessed using the method described by Shrout and Fleiss (Supplementary Appendix D).

## 3 Results

A total of 128 patients who underwent orthognathic surgery between September 2014 and December 2019 were included in the study. For 59 (46.1%) patients, operated from September 2014 until a transitional period between April 2016 and July 2016, analog planning was performed preoperatively using the 3D-OSS Articulator. Digital planning with the IPS CaseDesigner was carried out for the remaining 69 (53.9%) patients from April 2016 to December 2019. With a total of 84 (65.6%), most patients underwent bimaxillary surgery while the remaining 44 (34.4%) patients had only monognathic interventions. Of these, 27 patients had only Le Fort I maxillary osteotomy and 17 had a bisagittal split osteotomy alone. A significant difference between analog and digital planned interventions could only be observed regarding the frequency of an additional median sagittal split of the maxilla during Le Fort I osteotomy (Table 2).

**Table 2:** Characterization of 128 patients undergoing orthognathic surgery between September 2014 and December 2019.

Variable	Outcome	Analog	Digital	Total	*p*
		*n* = 59	*n* = 69	*n* = 128	
Sex	Male	24 (40.7%)	35 (50.7%)	59 (46.1%)	0.2887
	Female	35 (59.3%)	34 (49.3%)	69 (53.9%)	
Age at surgery	Mea*n* ± SD	24.12 ± 9.43	25.36 ± 8.48	24.79 ± 8.92	0.0810
	Median [1st Q./3rd Q.]	21 [18/25.5]	22 [19/30]	22 [19/27]	
Angle class	Class I	0 (0.0%)	3 (4.4%)	3 (2.3%)	0.1451
	Class II	26 (44.1%)	22 (31.8%)	48 (37.5%)	
	Class III	33 (55.9%)	44 (63.8%)	77 (60.2%)	
Bimax. surgery	Yes	37 (62.7%)	47 (68.1%)	84 (65.6%)	0.5777
	No	22 (37.3%)	22 (31.9%)	44 (34.4%)	
Median palate split	Yes	17 (28.8%)	8 (11.6%)	25 (19.5%)	0.0240
	No	42 (71.2%)	61 (88.4%)	103 (80.5%)	
Frontal open bite	Yes	22 (37.3%)	20 (29.0%)	42 (32.8%)	0.3494
	No	37 (62.7%)	49 (71.0%)	86 (67.2%)	

Analog and digitally planned interventions differed significantly only in terms of the frequency of an additional median sagittal split of the maxilla during Le Fort 1 osteotomy, which had been performed more frequently in previous years.

### 3.1 Results of Brons-Mulié analysis

**Figure 5:**
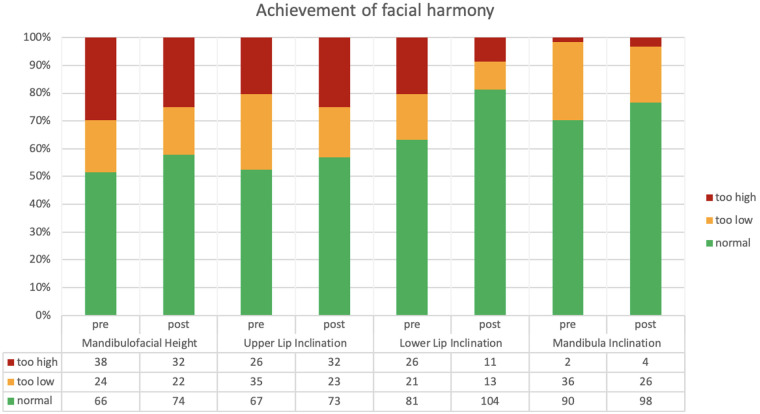
Achievement of facial harmony of the four Brons-Mulié parameters pre- and postoperatively (11). The green areas show the percentage of facial harmony. All four parameters show that facial harmony is achieved more frequently as a result of surgery (post).

**Figure 6:**
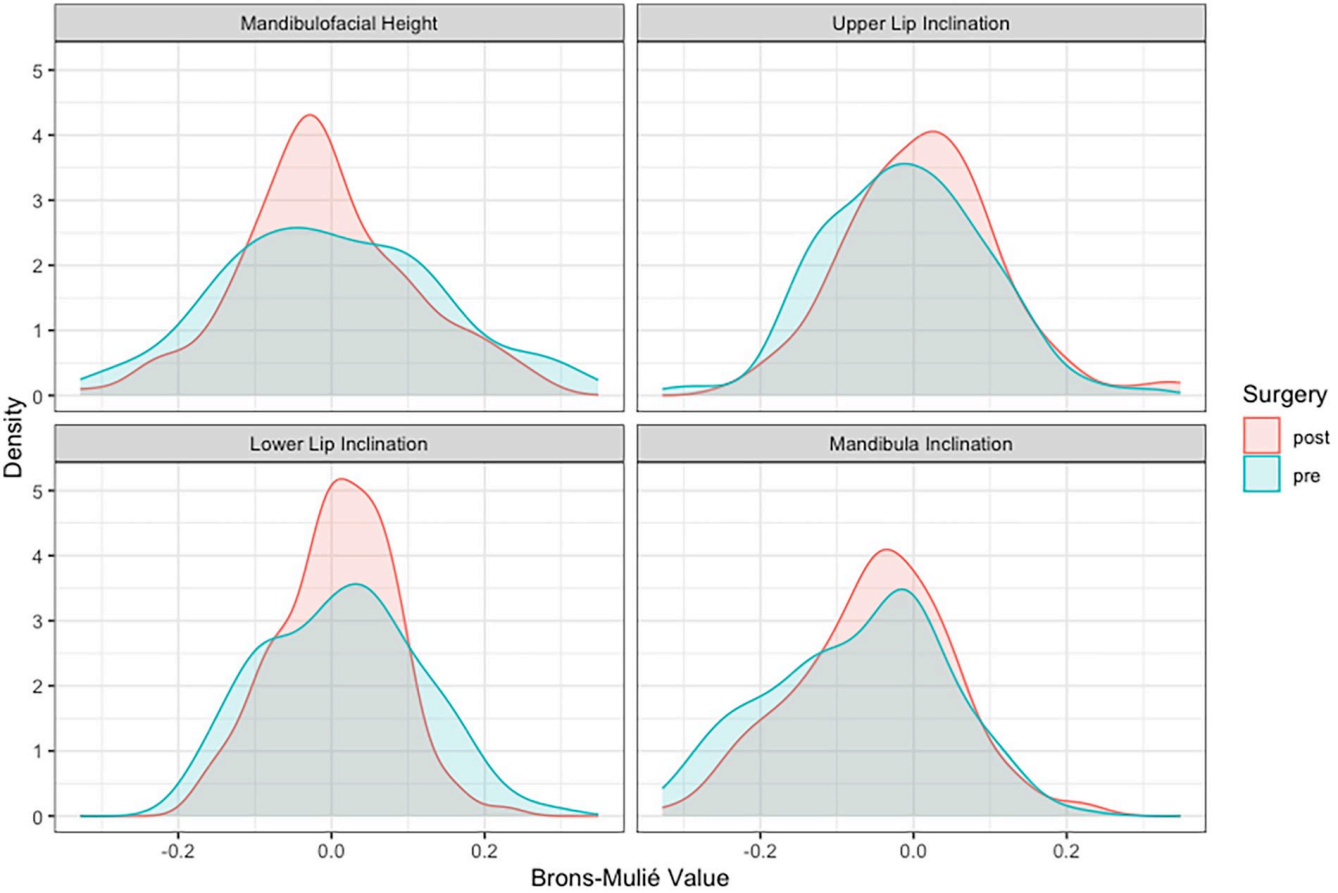
Density plots of the four transformed Brons-Mulié parameters: the graphs demonstrate a considerable modification of the curve-shape with a reduction in variance as a result of surgery. The changes of the Mandibulofacial Height and the Lower Lip Inclination were clearly visible (Table 3). Upper Lip and Mandibula Inclination showed an improvement in particular in anterior direction (positive values).

The preoperative application of Brons-Mulié Analysis was used in order to transfer patients' profile line into aesthetically favorable corridors as a result of surgery. An improvement could be shown for all four parameters of this analysis, as the corridors were achieved more frequently by the profile line after surgery (Figure 5). Unfortunately, as previously described (11), the improvement was less remarkable than expected. Thus, the corridor of facial harmony was achieved postoperatively in 74 (57.8%) of 128 cases for mandibulofacial height, in 73 (57.0%) cases for upper lip inclination, in 104 (81.3%) cases for lower lip inclination and in 98 (76.6%) for mandibular inclination (Figure 5). This represents an improvement of only a few percentage points for each parameter. In contrast, the above-described transformation of parameters into continuous variables showed a noticeable change as a result of surgery. Density plots of these parameters presented a definite reduction in postoperative variance compared to the preoperative variance (Figure 6) with a stronger accumulation of values around the means. A pronounced decline of variances was observed in mandibulofacial height and lower lip inclination (Table 3).

**Table 3:** Centrality, extreme values (Min. and Max.) and Variance (Var.) of the fifteen applied parameters.

Parameter	Surg.	Min.	1st Q.	Median	Mean	3rd Q.	Max.	Var.	F (p)	FK (p)
Brons-Mulié analysis:										
Mandibulofacial height	Pre	−0.3280	−0.1032	−0.0095	−0.0006	0.0967	0.3346	0.0198	0.0080	0.0016
	Post	−0.3280	−0.0813	−0.0238	−0.0082	0.0586	0.2676	0.0123		
Upper lip inclination	Pre	−0.3132	−0.0897	−0.0163	−0.0113	0.0536	0.3078	0.0107	0.8012	0.5223
	Post	−0.2373	−0.0509	0.0155	0.0130	0.0731	0.3484	0.0102		
Lower lip inclination	Pre	−0.1921	−0.0704	0.0195	0.0130	0.0845	0.2936	0.0103	0.0005	0.0004
	Post	−0.1708	−0.0396	0.0125	0.0065	0.0575	0.2258	0.0055		
Mandibula inclination	Pre	−0.3203	−0.1570	−0.0541	−0.0726	0.0026	0.2065	0.0130	0.1792	0.0690
	Post	−0.3203	−0.1129	−0.0453	−0.0506	0.0173	0.2194	0.0102		
Nakamura index:										
AI Nasal wing (h)	Pre	0.0004	0.0203	0.0442	0.0550	0.0804	0.2667	0.0019	0.0161	0.0121
	Post	0.0008	0.0183	0.0342	0.0427	0.0565	0.1745	0.0013		
AI Lip angle (h)	Pre	0.0026	0.0283	0.0488	0.0574	0.0758	0.1835	0.0017	0.0002	0.0079
	Post	0.0007	0.0136	0.0323	0.0382	0.0546	0.1464	0.0009		
AI Earlobe (h)	Pre	0.0003	0.0165	0.0315	0.0354	0.0480	0.1349	0.0006	0.5051	0.1805
	Post	0.0001	0.0123	0.0231	0.0286	0.0383	0.1167	0.0005		
AI Jaw Angle (h)	Pre	0.0003	0.0153	0.0330	0.0384	0.0594	0.2004	0.0009	0.0496	0.0076
	Post	0.0003	0.0131	0.0253	0.0315	0.0412	0.1302	0.0007		
AI Nasal wing (v)	Pre	0.0000	0.0063	0.0131	0.0139	0.0203	0.0541	0.0001	0.3915	0.4948
	Post	0.0000	0.0063	0.0118	0.0138	0.0182	0.0445	0.0001		
AI Lip angle (v)	Pre	0.0000	0.0048	0.0086	0.0112	0.0164	0.0363	0.0001	0.6082	0.3666
	Post	0.0000	0.0039	0.0080	0.0097	0.0131	0.0400	0.0001		
AI Earlobe (v)	Pre	0.0000	0.0049	0.0137	0.0178	0.0264	0.0715	0.0003	0.6251	0.4062
	Post	0.0000	0.0091	0.0158	0.0204	0.0255	0.0897	0.0003		
AI Jaw Angle (v)	Pre	0.0000	0.0082	0.0159	0.0183	0.0253	0.0752	0.0002	0.7698	0.5384
	Post	0.0000	0.0059	0.0143	0.0179	0.0266	0.0684	0.0002		
Enface analysis:										
Chin deviation	Pre	−0.3080	−0.0510	0.0260	0.0318	0.0983	0.5150	0.0182	0.0000	0.0000
	Post	−0.1630	−0.0080	0.0185	0.0292	0.0640	0.4840	0.0057		
Occlusal-plane inclination	Pre	−1.0940	−0.0760	0.0495	0.0417	0.1643	0.5650	0.0472	0.0003	0.0616
	Post	−0.5220	−0.0743	0.0175	0.0208	0.1050	0.3940	0.0248		
Midline shift	Pre	−0.5995	−0.2174	−0.0937	−0.0832	0.0456	0.5258	0.0435	0.0056	0.0236
	Post	−0.5425	−0.1306	−0.0642	−0.0481	0.0561	0.3442	0.0265		

Median and mean value of Brons-Mulié analysis and Enface analysis parameters fluctuate around zero. Median and mean value of Nakamura Index parameters lay above zero due to the absolute value function. Parametric *F*-test [F(p)] and non-parametric Fligner-Killeen test [FK(p)] were performed to compare the pre- and postoperative Variances (Var.). Multiplicity was addressed using false discovery rate (FDR) correction according to Benjamini–Hochberg (Table 4).

### 3.2 Results of Nakamura asymmetry Index

**Figure 7:**
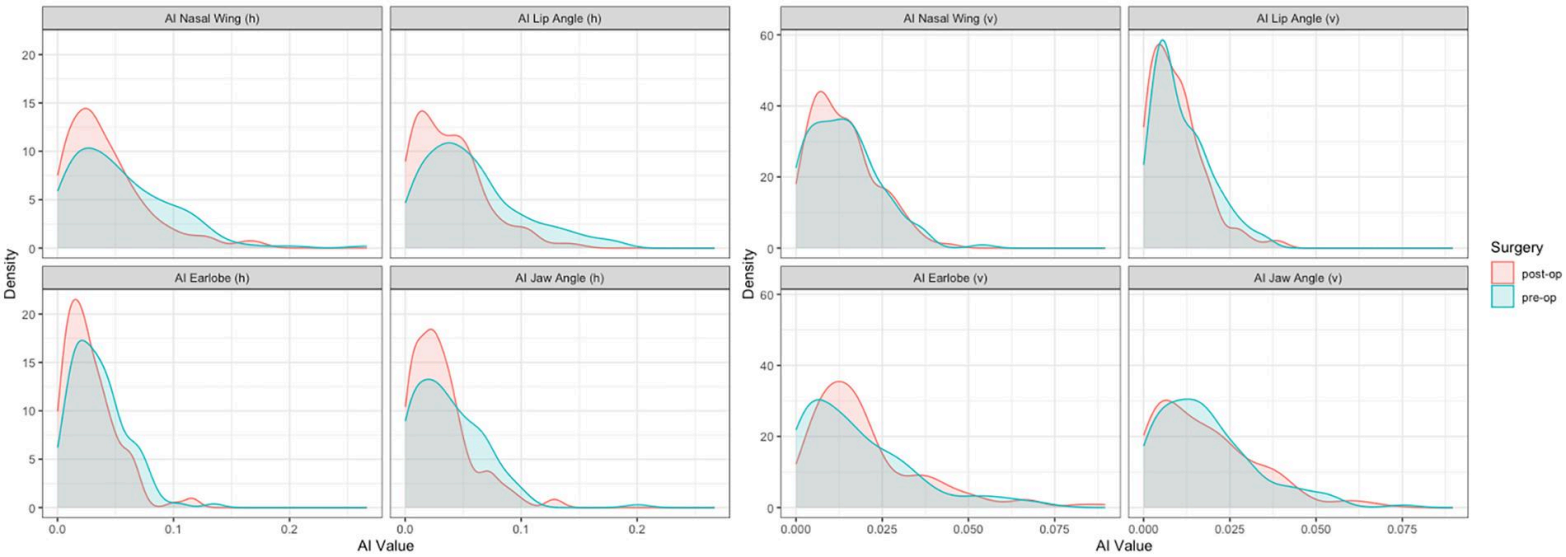
Density plots of the nakamura asymmetry Index (AI) in the horizontal **(h)** and vertical **(v)** dimension. A better symmetry was observed after orthognathic surgery in the horizontal dimension with a decreased variance. The results were significant regarding the parameters **an** [AI Nasal wing **(h)**], **ch** [AI Lip angle **(h)**] and **m** [AI Jaw Angle **(h)**]. In the vertical dimension the curves showed rather low AI values without a significant change in variance due to orthognathic surgery.

The Nakamura Asymmetry Index was determined to describe the symmetrization of faces by surgery. Using the absolute value function, the values were exclusively positive with a range from zero to a maximum of 0.2667. The median of the AI values measured in the horizontal dimension was 0.0383 preoperatively and could be reduced to 0.0281 postoperatively by orthognathic surgery. There was a pronounced decrease in variance for the pairs of points **an** [AI Nasal wing (h)], **ch** [AI Lip angle (h)] and **m** [AI Jaw Angle (h)] as a result of surgery (Table 3). In the vertical dimension, the measured AI values were predominantly smaller than in the horizontal dimension, and the resulting pre- and postoperative median values differed only slightly, with values of 0.0128 (preoperative) and 0.0122 (postoperative). In addition, no reduction in variance could be demonstrated in the vertical dimension (Table 3). Figure 7 shows the density plots of the AI values.

### 3.3 Results of Enface analysis

**Figure 8:**
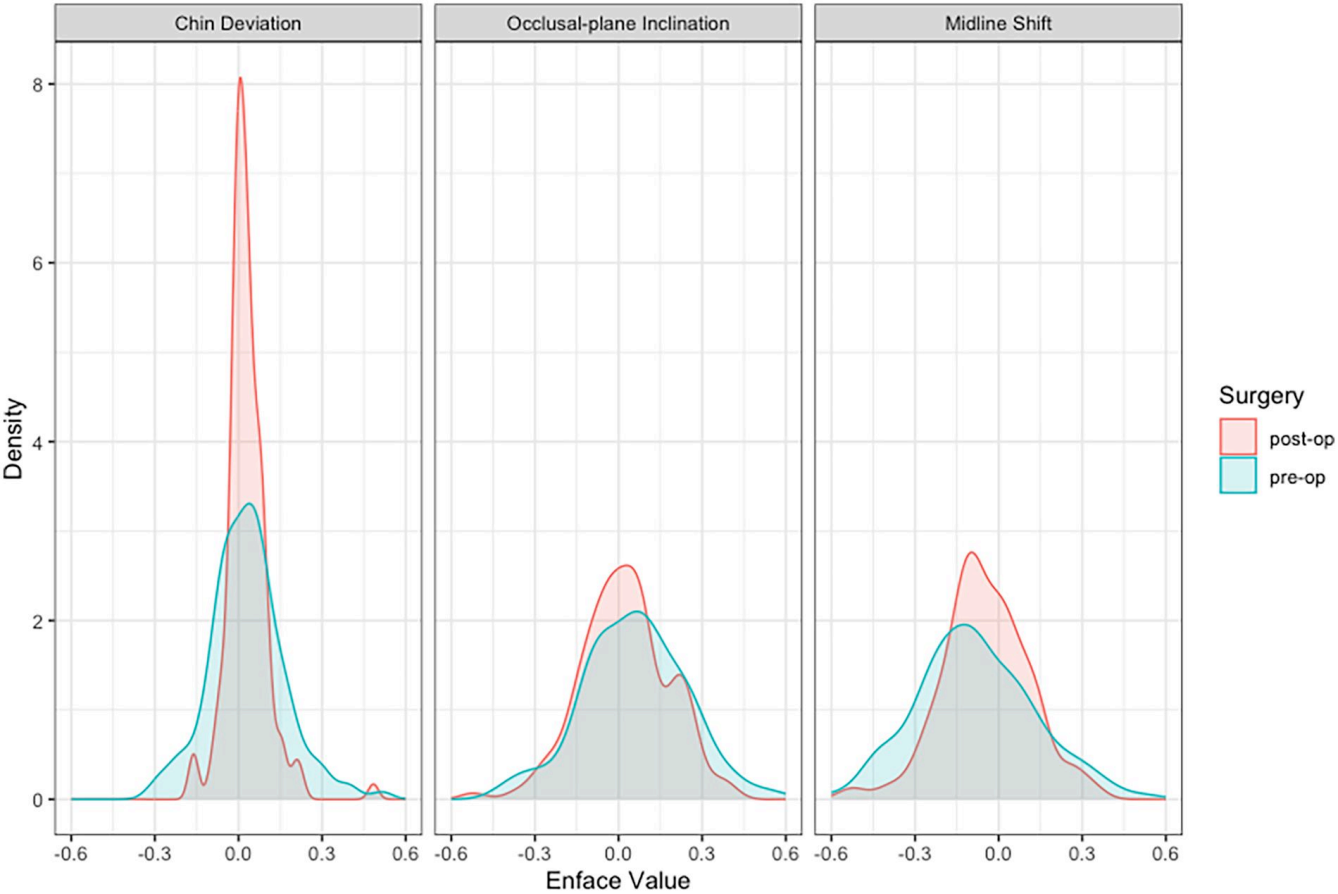
Density plots of the parameters of the enface analysis. A better symmetry could be observed after orthognatic surgery with significant reduced variances.

The parameters of the enface analysis are of fundamental importance in planning orthognathic surgery. The median of the transformed Chin deviation, which was measured as the angle to the median sagittal line across the Nasion point, was close to zero pre- and postoperatively (Table 3). The variance could be reduced from 0.0182 pre- to 0.0057 postoperatively as a result of orthognathic surgery (Figure 8). The median of the transformed occlusal-plane inclination was also approximately zero, but the variances were slightly higher at 0.0472 pre- and 0.0248 postoperatively. The transformed midline shift also fluctuated around zero pre- and postoperatively (Figure 8). The variance of the midline shift could also be reduced from 0.0435 to 0.0265 by orthognathic surgery. Overall, the reduction in variance was significant for all parameters of the enface analysis (Table 3, Figure 8).

### 3.4 Evaluation method

The transformed parameters of the three methods can be clearly summarized in one evaluation method (Table 3). Supplementary Figure A1 presents the medians and the measures of dispersion using boxplots. Inter- and intra-observer reliability for the landmarks used was satisfactory, showing a good level of reliability with ICC values above 0.9 for the majority of parameters (Supplementary Appendix D).

Multiplicity was addressed using false discovery rate (FDR) correction according to the Benjamini–Hochberg procedure (Table 4). The variance ratios [Var.(post)/Var.(pre)] were predominantly below the value of 1, indicating generally reduced postoperative variability. Among the statistically significant differences prior to adjustment, only the parameter AI jaw angle (h) showed a loss of significance when considering the *F*-test. When considering the FK-test, only the variance difference of the Midline Shift parameter was no longer statistically significant after FDR correction. The preoperative and postoperative variances, variance ratios, and corresponding 95% confidence intervals are presented in Table 4.

**Table 4:** Variance ratio and multiplicity analysis using false discovery rate (FDR) control according to Benjamini–Hochberg.

Parameter	Var. (pre)	Var. (post)	Var. ratio	95% CI lower	95% CI upper	F-Test (p)	F-Test FDR (p)	FK-Test (p)	FK-Test FDR (p)
Brons-Mulié analysis:									
Mandibulofacial height	0.0198	0.0123	0.62	0.44	0.88	0.0080	0.0199	0.0016	0.0081
Upper lip inclination	0.0107	0.0102	0.96	0.67	1.36	0.8012	0.8012	0.5223	0.5384
Lower lip inclination	0.0103	0.0055	0.54	0.38	0.76	0.0005	0.0019	0.0004	0.0027
Mandibula inclination	0.0130	0.0102	0.79	0.56	1.12	0.1792	0.2987	0.0690	0.1150
Nakamura index:									
AI Nasal wing (h)	0.0019	0.0013	0.65	0.46	0.92	0.0161	0.0345	0.0121	0.0304
AI Lip angle (h)	0.0017	0.0009	0.52	0.36	0.73	0.0002	0.0017	0.0079	0.0236
AI Earlobe (h)	0.0006	0.0005	0.89	0.63	1.26	0.5051	0.6888	0.1805	0.2707
AI Jaw Angle (h)	0.0009	0.0007	0.70	0.50	1.00	0.0496	0.0931	0.0076	0.0236
AI Nasal wing (v)	0.0001	0.0001	0.86	0.61	1.22	0.3915	0.5872	0.4948	0.5384
AI Lip angle (v)	0.0001	0.0001	0.91	0.64	1.29	0.6082	0.7212	0.3666	0.4999
AI Earlobe (v)	0.0003	0.0003	1.09	0.77	1.55	0.6251	0.7212	0.4062	0.5078
AI Jaw Angle (v)	0.0002	0.0002	1.05	0.74	1.49	0.7698	0.8012	0.5384	0.5384
Enface analysis:									
Chin deviation	0.0182	0.0057	0.31	0.22	0.44	0.0000	0.0000	0.0000	0.0000
Occl.-plane inclination	0.0472	0.0248	0.53	0.37	0.75	0.0003	0.0017	0.0616	0.1150
Midline shift	0.0435	0.0265	0.61	0.43	0.86	0.0056	0.0169	0.0236	0.0505

Variance ratios (Var. ratio = Var.post/Var.pre) are presented to quantify changes in dispersion following orthognathic surgery. Smaller ratio values indicate a greater reduction in variance and may therefore suggest a stronger surgical effect. Ninety-five percent confidence intervals are provided for all variance ratios. *P*-values were adjusted for multiple comparisons using false discovery rate (FDR) control according to the Benjamini–Hochberg procedure.

**Figure 9:**
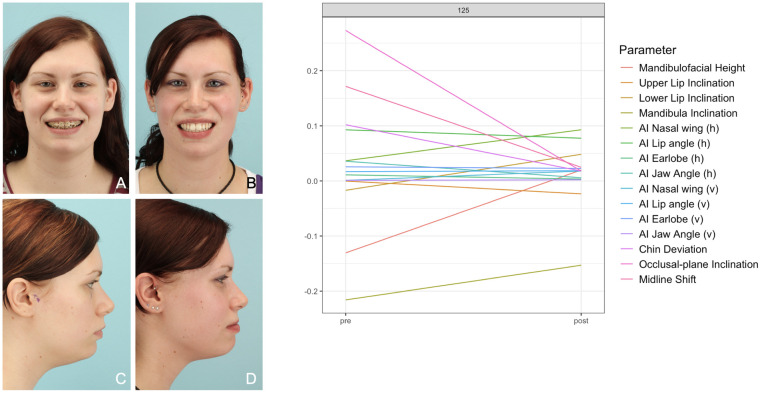
Patient No. 125 with Class II malocclusion. The diagram shows a mainly convergent pattern of straight lines due to a nice and symmetric postoperative result. **(A,C)** preoperative photos; **(B,D)** postoperative photos.

**Figure 10:**
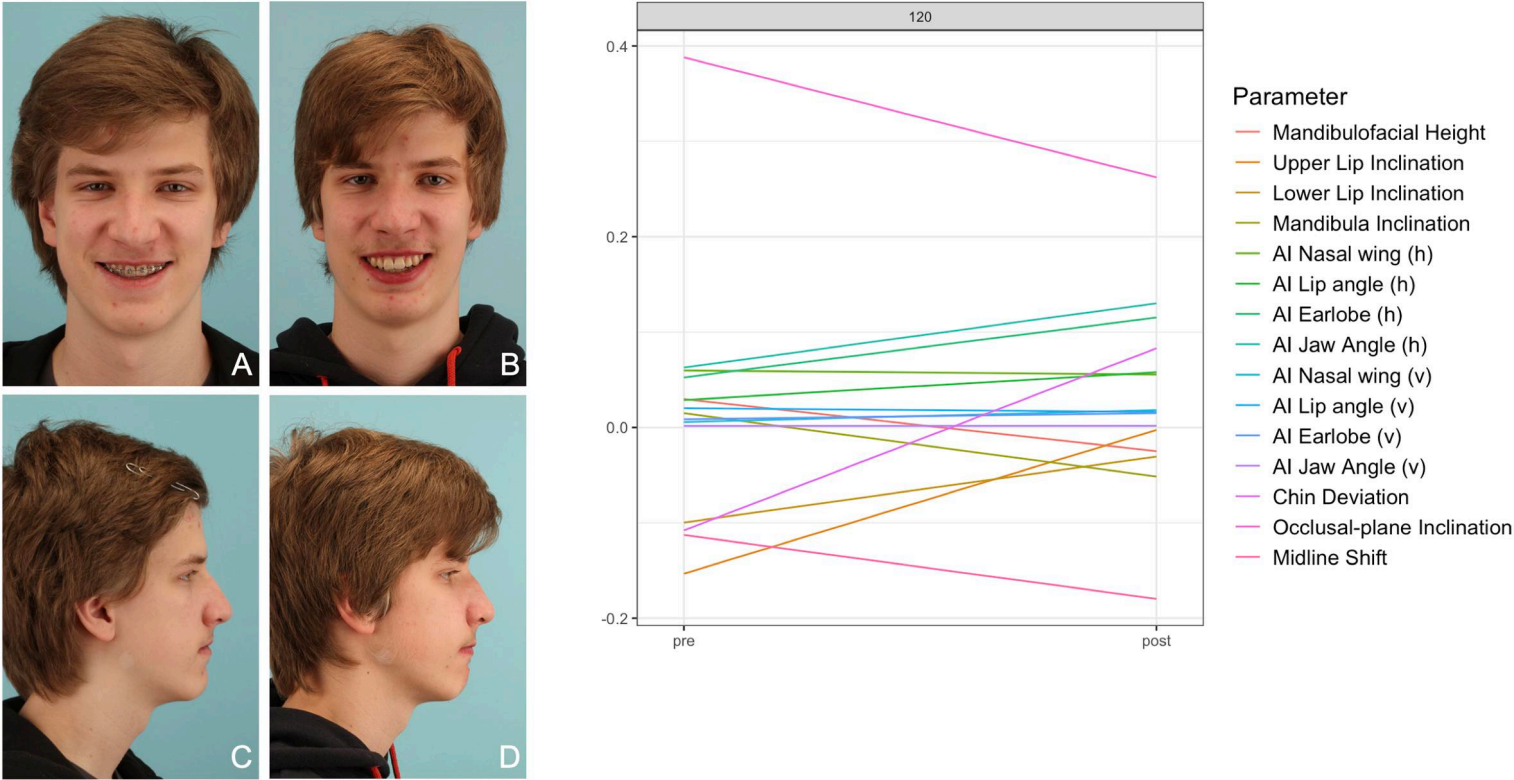
Patient No. 120 with Class III malocclusion. The diagram shows a partially divergent pattern of straight lines. Overall postoperative result is appealing but symmetry could be improved. **(A,C)** preoperative photos; **(B,D)** postoperative photos.

**Figure 11:**
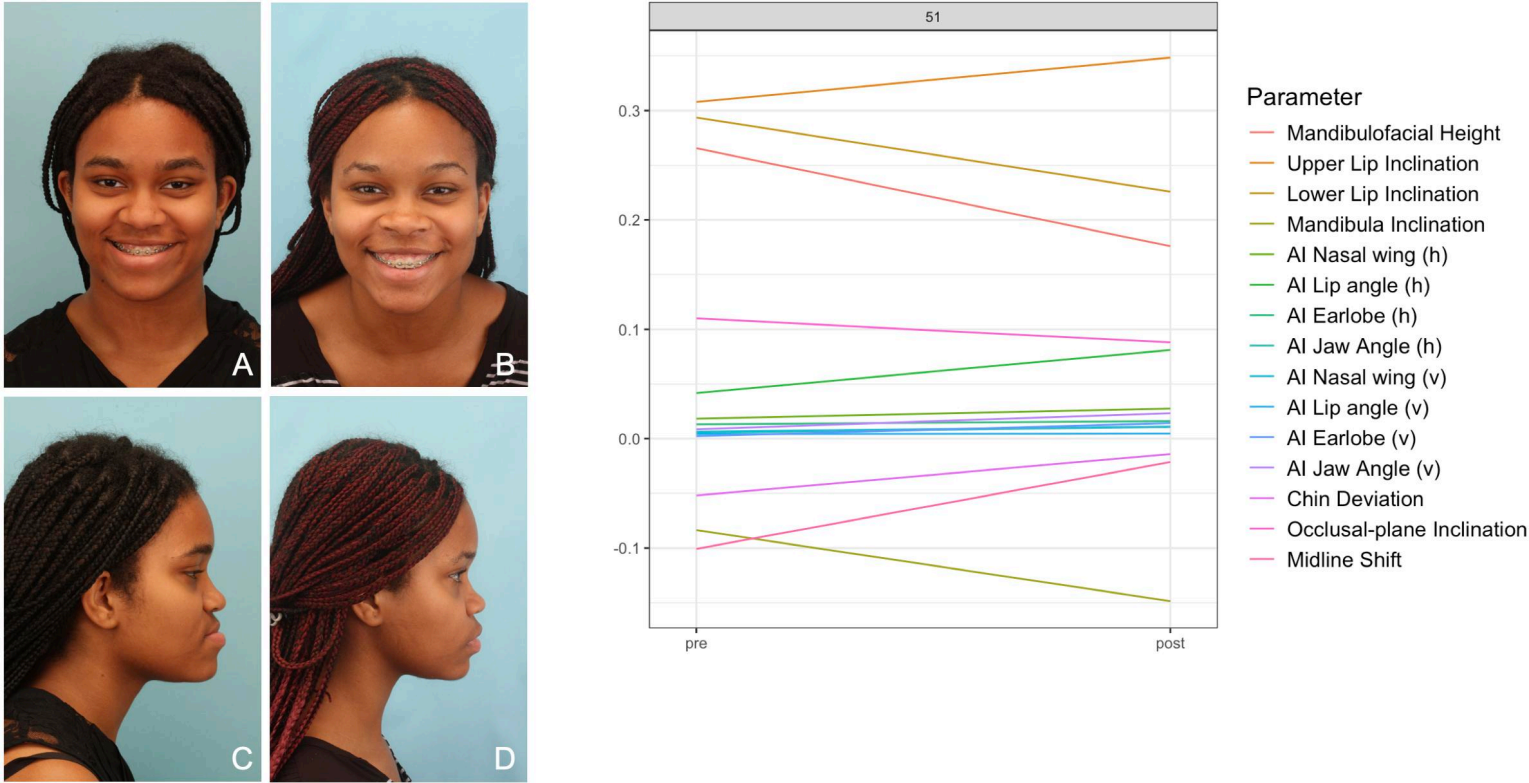
Patient No. 51 with Class III malocclusion. The diagram shows a significant improvement and a proper symmetry but a persistent postoperative long face constellation with a ventral characteristic. **(A,C)** preoperative photos; **(B,D)** postoperative photos.

**Figure 12:**
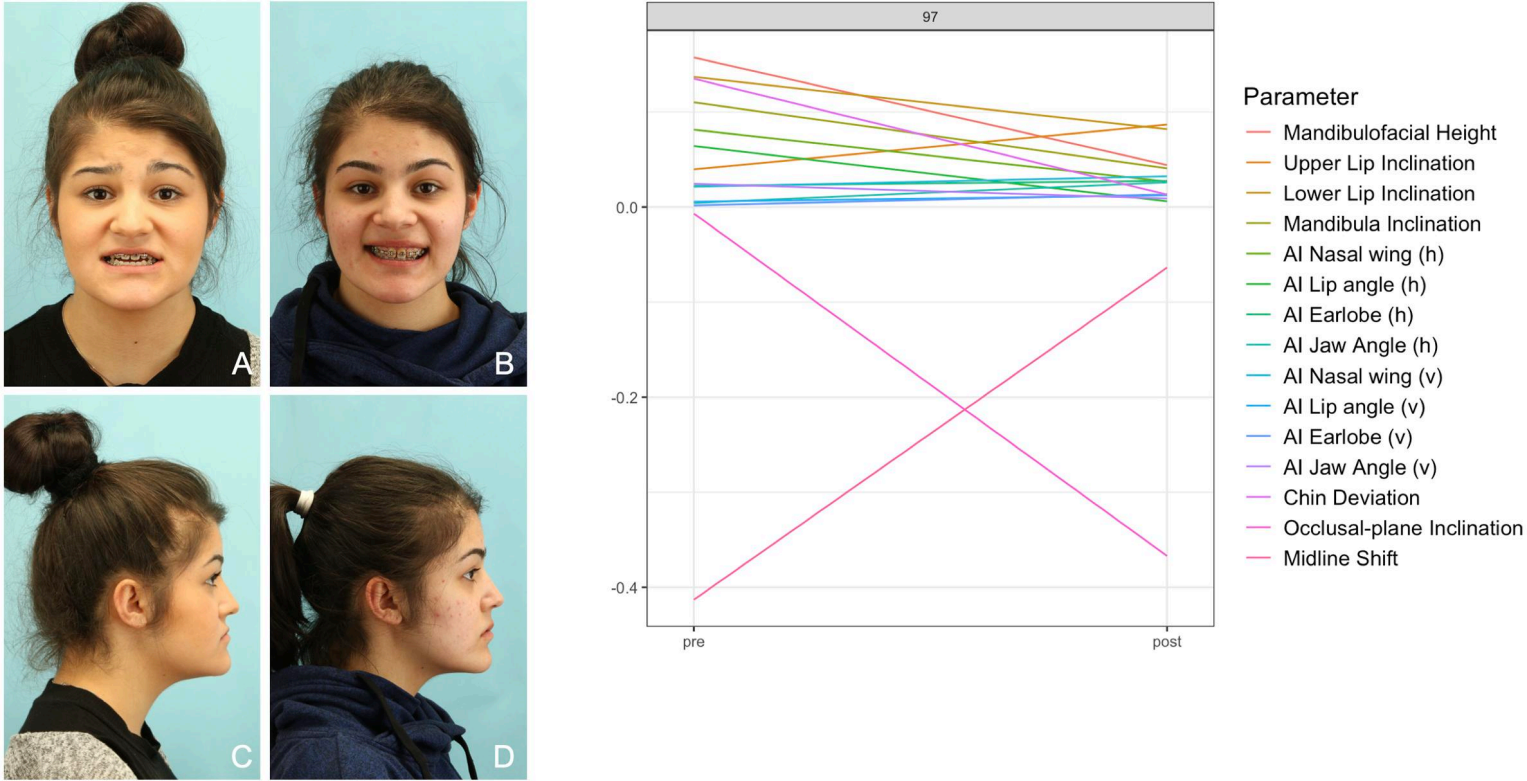
Patient No. 97 with Class III malformation. The diagram shows an extreme value postoperative due to the unfavorable canted occlusal plane. Revision surgery was necessary. **(A,C)** preoperative photos; **(B,D)** postoperative photos.

Supplementary Figure A2 displays all 15 parameters for each patient as straight lines, allowing a graphical evaluation for all 128 patients simultaneously. If the straight lines move towards the zero line (convergent pattern), it can be assumed that orthognathic surgery has improved symmetry and aesthetics (Figure 9). A separation of the straight lines from zero, on the other hand, indicates a deterioration (divergent pattern) (Figure 10). Further examples are shown in Figures 11 and 12.

### 3.5 Comparision between digital and analog planning

As an exploratory analysis, postoperative and preoperative variances were compared between analog and digitally planned surgeries to assess potential differences between planning techniques. While no differences between digitally and analogically planned surgeries were found preoperatively, a significant difference was postoperatively only detected for the point **ado** (earlobe) in the vertical dimension (Supplementary Table B1). No consistent or systematic advantage of digital planning was observed across the remaining parameters. A closer examination of the point **ado** in the vertical dimension [earlobe (v)] revealed a strongly left-skewed distribution of the data. In this context, the non-parametric Fligner-Killeen indicated a marginally significant difference in favor of digital planning. Postoperative variances stratified by planning technique are summarized in Supplementary Table B1.

## 4 Discussion

### 4.1 2D analysis for objective description of the aesthetic appearance of the face

The aim of orthognathic surgery should not only consist in achieving a stable and neutral occlusion, but also in creating a good aesthetic appearance of the face. There is clear evidence that symmetry and averageness are important aspects of the attractiveness of the human face (5, 13, 21–24). Langlois et al. were able to show that faces that were digitally composed of many individual faces were judged to be most attractive in the end (25, 26). They were also able to show that attractiveness increased the more features were adjusted in the direction of more averageness (25). As described by Berlin et al., symmetry of the face also represents a form of averageness, suggesting that increasing facial symmetry can also increase the attractiveness of the face (5). However, averageness is not only important in the frontal view but also in the lateral view of the facial profile (5). Gu et al. demonstrated that the lateral attractiveness of the face correlates well with the frontal attractiveness of the face (27) and Spyropoulos et al. reported that an average profile line also generates the highest attractiveness in the lateral view of the head (28). Canut et al. postulated that the balance of the three prominent facial features, the nose, lips and chin, also plays an essential role in achieving beauty in the human face (29). In this context, Brons and Mulié introduced the concept of facial harmony (8). They developed a method based on the golden ratio to determine a harmonious and attractive shape of the facial profile line, allowing the lateral aesthetics of the face to be mathematically objectified (8, 11). Such a profile analysis of the soft tissues can be usefully combined with an enface-analysis of prominent features in the frontal view (5) and an analysis of facial symmetry, for instance with Nakamura's asymmetry index (5, 14). In this way, the aesthetic appearance of the face can be described using objective methods.

Since orthognathic surgery can significantly influence the shape of the face in all three dimensions, it seems absolutely advisable to carry out a precise analysis of the face with regard to averageness, the symmetry and the shape of the profile line prior to any orthognathic surgical procedure (5, 9, 11). 2D analyses from different views, such as lateral cephalometry, have proven their worth in orthodontics and orthognathic surgery for many years (5, 30). With the help of normal value corridors, skeletal malformation is described and therapy is directed towards a change in the direction of the normal, which means in other words also in the direction of the averageness (31, 32). The analysis of standardized 2D photos from different perspectives has also proven its worth in this context (6, 16). Thus, profile photographs can be easily analyzed using the Brons-Mulié method with regard to the contour of the profile line without using any x-rays (8, 11). The normal value corridors specified by Brons and Mulié can be very useful for analyzing the profile line with regard to facial harmony (10). In the frontal view, symmetry can be described using Nakamura's asymmetry index as well as a simple enface analysis can be applied to determine the parallelism of the occlusal plane with the inter-pupillary line, the deviation of the chin and the midline shift (5). Overall, the combination of the mentioned 2D analyses can be very useful to optimize treatment planning in terms of symmetry, averageness and facial harmony, as they are able to show the proper orientation of the necessary movements during the planning procedure in orthognathic surgery (5). The application of the well-proven 2D analyses also follows the recommendation of Margolis et al. (33): Division of the face and dentition into separate dimensions (transverse, vertical, anteroposterior) can be a useful way of systematically breaking down a complex problem into its component parts so that the optimal treatment plan can be developed for a given patient (33).

However, the results can also be of great benefit when using modern digital 3D planning software, as three-dimensional cephalometry and 3D surgical simulation is highly dependent on the correct setting of the natural head position in the virtual 3D model (31, 34, 35). In this context, 2D analyses can be also very beneficial in determining the correct position of the head in virtual three-dimensional space (31, 36).

### 4.2 Quality assurance

Since orthognathic surgery can have a strong direct influence on symmetry and the profile line of the face, it is advisable to perform the 2D analyses mentioned above not only preoperatively but also postoperatively for quality assurance. A main question to be answered is whether and to what extent the preoperatively formulated goals could be achieved during the surgical intervention. With the open-source software used in this study, such a follow-up evaluation can be carried out easily and quickly on the basis of standardized photographs (6, 16). The data for this purpose can be generated automatically as a by-product, which can be used for such a comparison between the preoperative and the postoperative condition. Since it is often necessary to make compromises with regard to the normalization of all parameters during surgical planning, it makes sense to describe quality assurance not only by the binary variables of achieving normal value corridors (yes/no) but also by measuring the deviation from the optimum value. In this way, density plots can be used to describe the real changes that have been achieved in terms of symmetry, averageness and harmony. By analyzing the variances of the individual parameters, the change towards an attractive appearance can also be mathematically proven, despite the large variety of possible skeletal malformations. As shown in Table 3, significant reductions in variance were found for the parameters mandibulofacial height, lower lip inclination, AI nasal wing (h), AI lip angle (h), AI jaw angle (h), chin deviation, occlusal plane inclination and midline shift. The density plots also show a stronger accumulation around the optimum of zero for most of the other parameters postoperatively, even if this was not significant (Table 3). Only the vertical determinations of the asymmetry indices were obviously only insignificantly influenced by the surgical interventions.

Quality assurance is always a topic of great interest in orthognathic surgery. There are numerous studies on whether professionals, laypersons and patients rate the results of the surgical procedures performed as attractive and satisfactory (37–39). Although the majority of these studies come to positive conclusions, such assessments are always the result of subjective judgements. Objective methods, on the other hand, are used to investigate whether the proportions predicted by planning software could be accurately achieved by the surgical intervention (40). For instance, it is investigated whether the position and shape of the lips and chin following orthognathic surgery correspond exactly to the digital prediction (40). Such a simulation of the post-operative conditions can be extremely useful in the digital planning of orthognathic surgery, as it allows the post-operative appearance of the patient to be at least partially predicted visually. However, there is generally no guidance available for achieving the criteria of optimal facial attractiveness. In this context, the work on the aimed-at profile line published by Freihofer and Mooren in 1997 and 1998 is still of great importance (9, 10). In these studies, surgeons were asked to draw the best possible profile line (aimed-at profile) for previously completely unbalanced facial profiles according to their subjective points of view. In doing so, a large variability between the profile lines drawn by the different surgeons was shown, which in some cases also deviated strongly from the golden ratio advocated by Brons and Mulié (8). The authors therefore concluded that the subjective variability of the surgeons was actually too great for an orthognathic surgical procedure and that objective methods should be used for planning (10). Although modern planning software for orthognathic surgery can contribute a considerable amount to achieve the most possible symmetrical surgical results (41, 42), dedicated modules specifically designed to optimize postoperative facial aesthetics are usually not included. Furthermore, the software employed for preoperative planning is, in most cases, not reused postoperatively for quality assurance purposes, particularly because acquiring a repeat CT scan would expose the patient to additional radiation. The here presented method is based on established and published procedures and it allows a facile comparison between pre- and postoperative (5, 8). It is based on freeware tools and can be used to monitor a large number of orthognathic interventions without major financial and personnel outlay. The patient diagrams provide a good overview and can also help to easily identify problematic surgeries by searching for divergent patterns and extreme values. This can potentially help to detect and to correct systematic errors in preoperative planning or surgical implementation. Deriving the methodology from the criteria of an attractive face (symmetry, averageness, facial harmony) (13) also enables to draw objective conclusions about pre- and postoperative facial aesthetics and how they are influenced by orthognathic surgery.

### 4.3 Comparision between analog and digital planning

Until 2016, orthognathic surgery at Giessen University Hospital was planned analogously using the 3D OSS articulator, and from 2016 onwards, IPS Case Designer was used for digital planning. The question therefore arose as to whether the postoperative evaluation of the surgical interventions could reveal any differences between the two planning methods. As an exploratory assessment, preoperative variances of the 15 individual parameters were compared. Only the variance of the midline shift showed a slightly higher value for digitally planned interventions; otherwise, no relevant preoperative differences were observed (Supplementary Table B1). Postoperative variances similarly showed no significant differences for 14 out of 15 parameters (Supplementary Table B1). Due to the sequential implementation of the planning methods, comparisons are inherently limited. Nevertheless, as all other operational conditions were comparable, the data suggest that the choice of planning method did not substantially affect the outcomes. Both the analog planning method using the 3D OSS articulator and the digital method using the IPS Case Designer achieved comparable results overall. One parameter showed a significantly lower variance postoperatively after digital planning than following analog planning. This was the vertical asymmetry index of the point **ado** [AI Earlobe (v)] in the anterior view. This could be a coincidence, but it could also indicate a real effect of digital planning. During planning using the 3D-OSS articulator, the mandible was always aligned in such a way that the bony surfaces of the sagittal splits in the area of the jaw angles were as parallel to each other as possible (17). However, the symmetry of the jaw angles, especially in the projection from caudal onto the zygomatic arches, could not be checked in the articulator, as the bony structures of the midface could not be displayed simultaneously. Digital planning on the other hand allows this kind of control. The skull can be rotated three-dimensionally during the surgical simulation and viewed from all sides (34). The point ado is dependent on the position and symmetry of the jaw angle and it is therefore conceivable that digital planning offered a potential advantage in terms of facial symmetry. But overall, parameters were similar across planning techniques, and no systematic differences could be observed. Any apparent benefit of digital planning remains exploratory and should be interpreted with caution.

### 4.4 Advantages and disadvantages of the analysis method

The here presented method is based on basic two-dimensional standard photographs and can be carried out easily and free of charge using open-source software. However, it is important that the photos of the facial cranium are taken at high quality according to published standards and without distorting or skewing the skull, as otherwise inaccuracies may quickly arise with regard to the facial midline and the position of the chin (Supplementary Appendix C). It is also very beneficial if photos are taken from a standardized distance, for instance with a tripod at predefined object distances (6). In this way, negative side effects caused by different object scales can be reduced as much as possible (5, 6). However, as described by Berlin et al., further inaccuracies due to inter- and intra-observer variability must also be taken into account when defining points and lines on the photos (5). In particular, variability is to be expected for landmarks that must be positioned at the apex of a curvature, such as point m (jaw angle), or within the central area of a surface, such as the point Pogonion for the assessment of Chin deviation (Supplementary Appendix D). Here, the digital definition of points and lines on the screen may offer advantages over drawings on paper due to the possibility of instant correction. Despite the demonstration of satisfactory inter- and intraobserver reliability for the majority of parameters in this study (Supplementary Appendix D), targeted examiner training may further enhance measurement consistency. In cases of a significantly skewed natural head position or the presence of pronounced cranial asymmetries, it can also be challenging to draw a correctly aligned median-sagittal line, which can have consequences for all subsequent measurements. Ultimately, these inaccuracies cannot be completely avoided even not in advanced three-dimensional analysis software (43, 44). Furthermore, the general problem remains that no software is able to determine exactly how many millimeters skeletal components need to be moved in order to achieve a desired and clearly defined effect on the facial soft tissues (11). More important than accuracy, however, is that symmetry deficits and unfavorable contours of the profile line are properly identified and corrected in the right orientation during planning and performing orthognathic surgery (15). It is therefore even possible that a proper skeletal plan has to be corrected due to aesthetic needs in order to achieve an acceptable aesthetic result (45). The knowledge about the secrets of facial attractiveness, which can be derived from averageness, symmetry and facial harmony, is crucial in this context (8, 13). The method presented in this study may contribute to the objective characterization and refinement of facial aesthetics, thereby serving as a valuable complement to contemporary three-dimensional planning techniques. In summary, 2D analyses are sufficient for monitoring symmetry and facial harmony, whereas 3D imaging is most informative when precise spatial measurements or preoperative surgical planning require detailed geometric data (41, 42).

## 5 Conclusions

Orthognathic surgery should not only lead to a stable and neutral occlusion, but also contribute to an improvement in facial aesthetics. The method used in this study, which is based on well-established 2D analyses for standardized facial photos, allows subjective aesthetic appearance of the human face to be visualized in an objective procedure. It may therefore be used for preoperative surgical planning to complement modern three-dimensional planning techniques, as well as for postoperative quality assurance in orthognathic surgery. No evidence was found to indicate that digital planning offers a decisive advantage over conventional analog planning.

## Data Availability

The raw data supporting the conclusions of this article will be made available by the authors, without undue reservation.
