# Correction: Quality assurance in orthognathic surgery using Brons-Mulié's soft tissue analysis, Nakamura's asymmetry index and a simple enface analysis

**DOI:** 10.3389/froh.2026.1884329

**Published:** 2026-06-01

**Authors:** Frontiers Production Office

**Affiliations:** Frontiers Media SA, Lausanne, Switzerland

**Keywords:** Brons-Mulié analysis, enface analysis, facial harmony, facial photography, nakamura asymmetry index, orthognathic surgery

There was a mistake in Figure 2 as published. A blurred version was incorrectly used. The corrected Figure 2 appears below.

**Figure 2:**
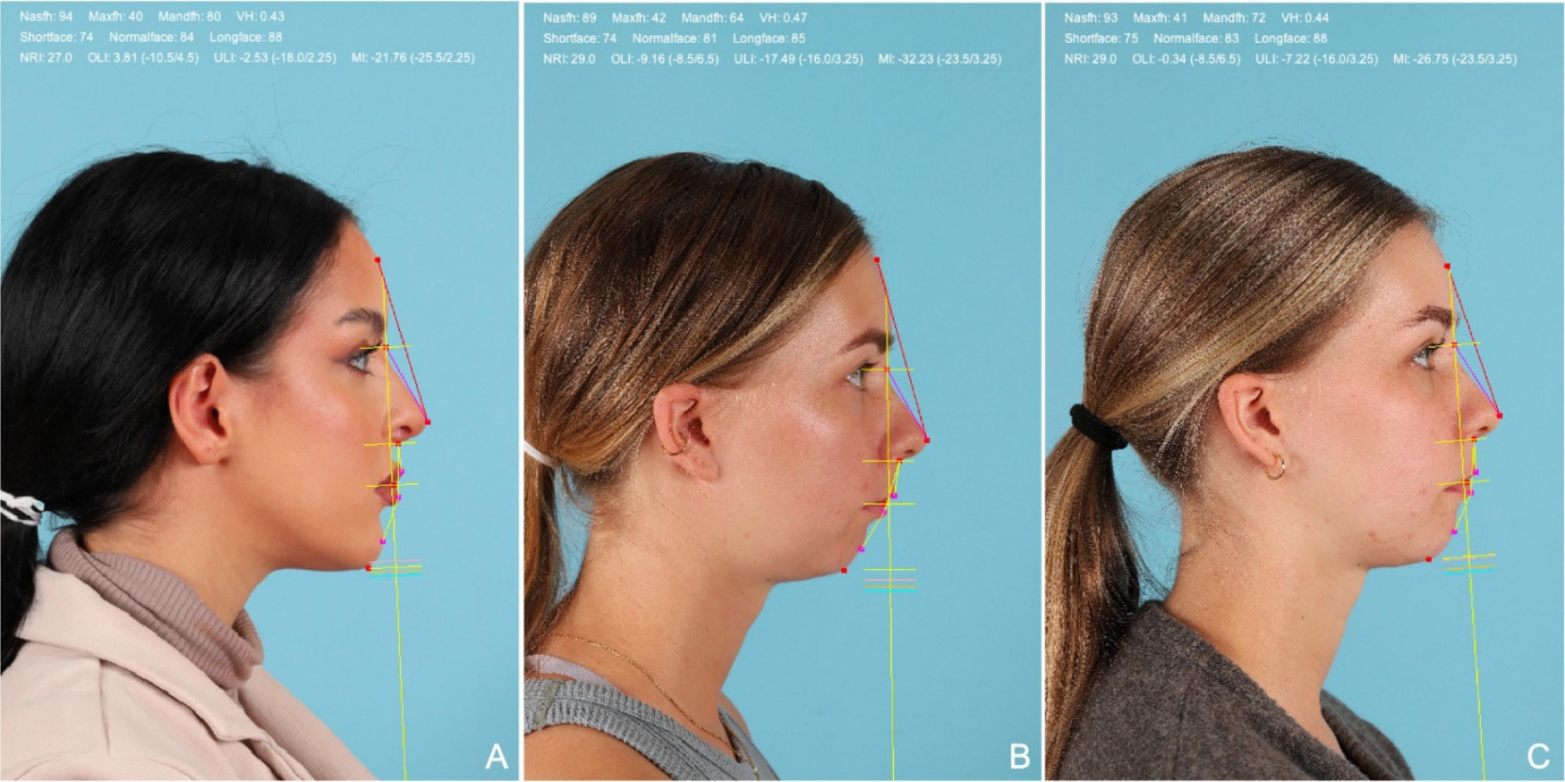
Profile analysis according to brons and mulié (**11**). Left photo **(A)** shows a profile with satisfactory facial harmony in vertical and sagittal dimensions. Right photos show the preoperative **(B)** and the postoperative **(C)** profile of a patient with Class II malocclusion. This profile could be significantly improved but was still not perfect after surgery, because the chin was still too short in both, the vertical and the sagittal dimension.

The original version of this article has been updated.

