# Supplementary material for: Quality assurance in orthognathic surgery using Brons-Mulié's soft tissue analysis, Nakamura's asymmetry index and a simple enface analysis
Source: Front Oral Health. 2026 Mar 26;7:1748425. doi: 10.3389/froh.2026.1748425 (PMC13062170; doi:10.3389/froh.2026.1748425)

Quality assurance in orthognathic surgery using Brons-Mulié's soft tissue analysis, Nakamura's asymmetry index and a simple Enface analysis

Appendix A: Graphical Presentation of the Data (Boxplots and Line Plots)

Figure A.1: Evaluation Method: Visualization of data distribution of all 15 parameters using boxplots. Postoperatively, most parameters show a reduced dispersion and a shift towards zero.

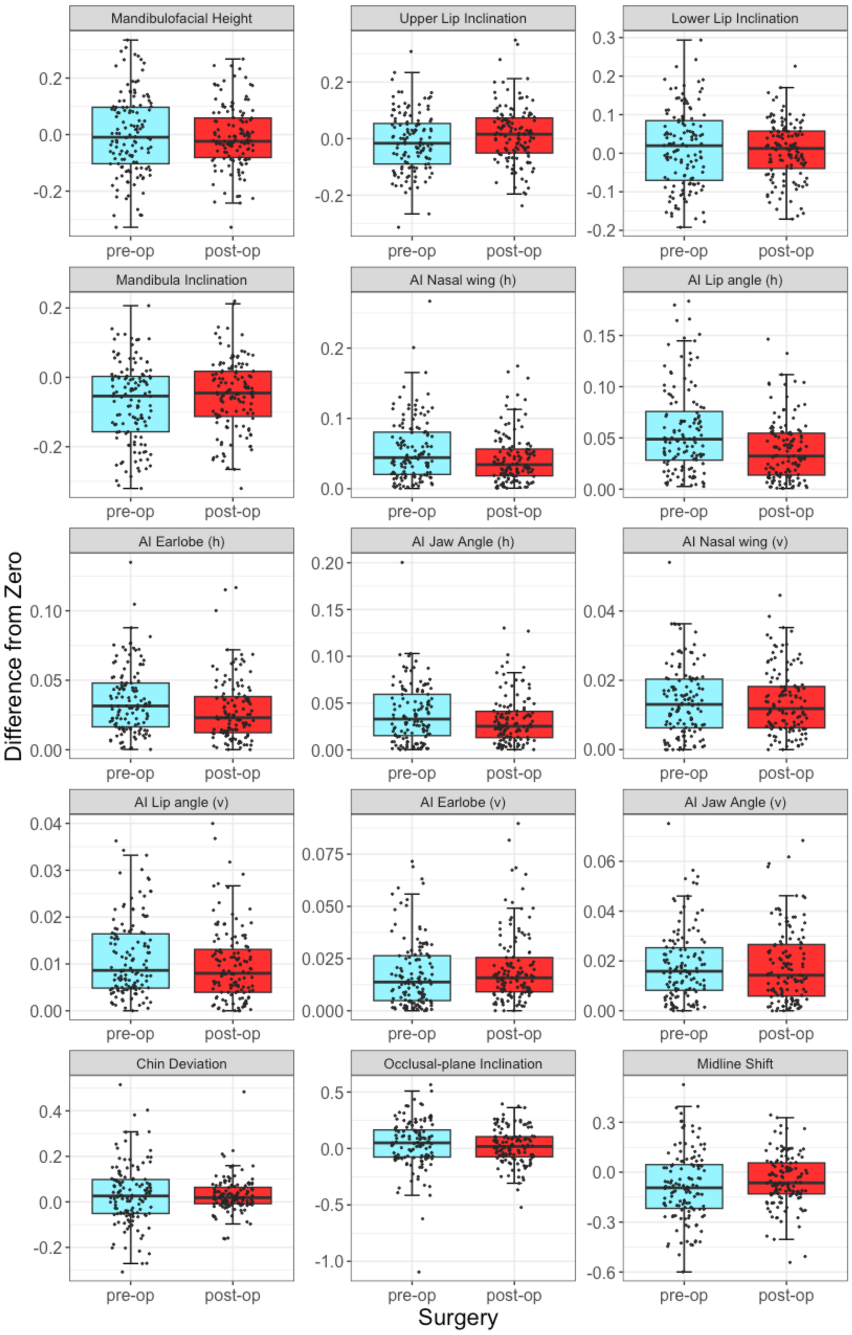

Figure A.2: Visualization of all 128 orthognathic interventions from pre- to postoperative using straight lines. A convergent pattern indicates good harmonization and symmetrization of the face. In contrast, a divergent pattern indicates deterioration in symmetry and aesthetics. Straight lines that deviate severely from zero indicate pre- or postoperative extremes. Two cases showed pre-operative values below -0.6, which are not plotted in this overview.

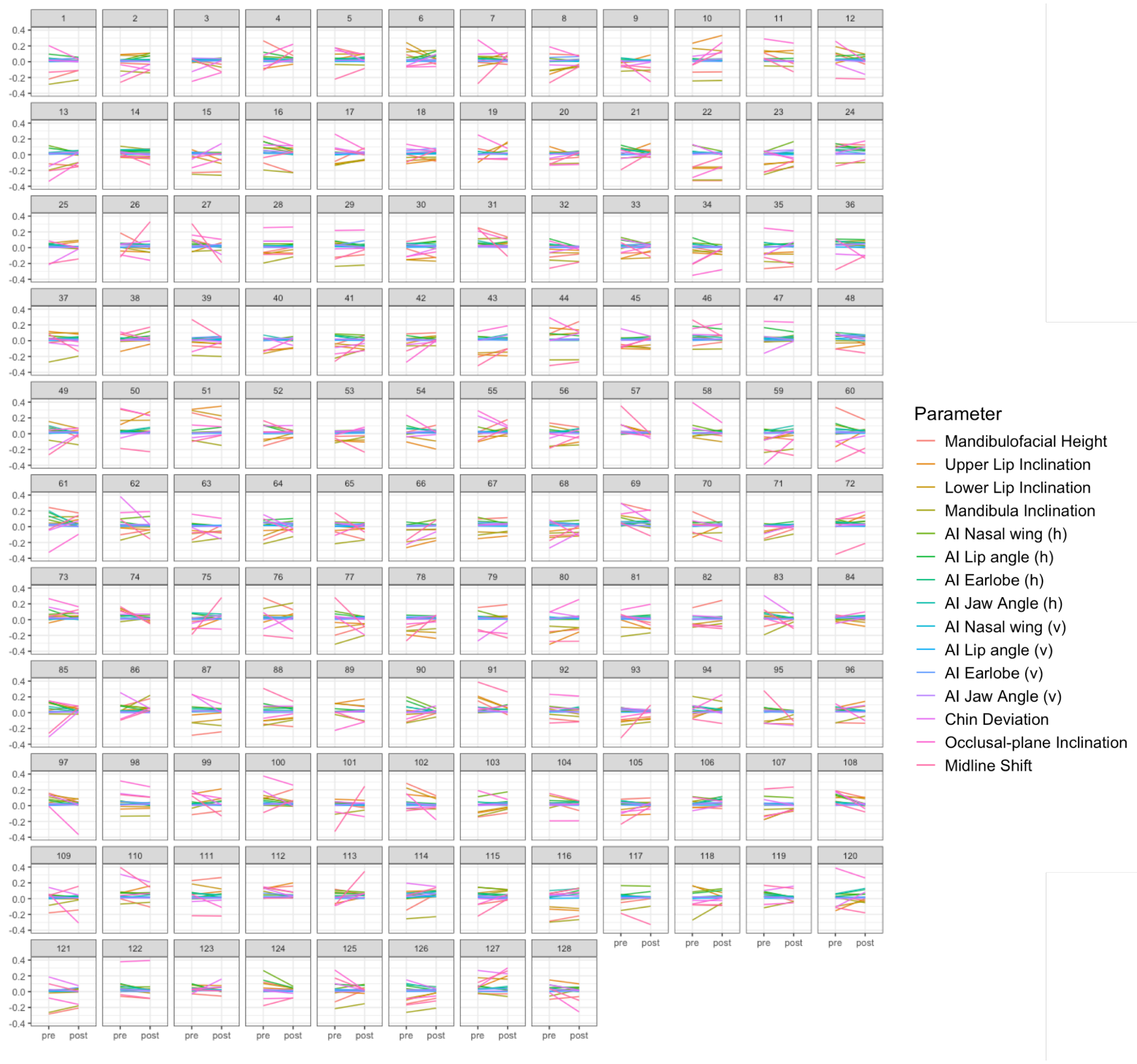

Appendix B: Exploratory Comparison Between Digital and Analog Planning

Table B.1: Exploratory Comparison between digital and analog planning. No significant differences between the variances were found preoperatively. Postoperatively, only the parameter AI Earlobe (v) showed a slightly significant difference in favor of digital planning. Otherwise, no significant difference in the variances could be determined as well.

	Var. (pre) analog	Var. (pre) digital	F (pre) (p)	FK (pre) (p)	Var. (post) analog	Var. (post) digital	F (post) (p)	FK (post) (p)
**Brons-Mulié Analysis:**								
Mandibulofacial Height	0.0169	0.0217	0.3339	0.1302	0.0103	0.0126	0.4342	0.2143
Upper Lip Inclination	0.0095	0.0117	0.4158	0.8626	0.0081	0.0121	0.1164	0.2047
Lower Lip Inclination	0.0088	0.0118	0.2671	0.1608	0.0055	0.0057	0.9155	0.7658
Mandibula Inclination	0.0146	0.0112	0.2865	0.1406	0.0116	0.0084	0.1948	0.2099
**Nakamura Index:**								
AI Nasal wing (h)	0.0022	0.0017	0.2994	0.2841	0.0016	0.0010	0.0916	0.1784
AI Lip angle (h)	0.0020	0.0015	0.2342	0.1557	0.0011	0.0006	0.0144	0.0927
AI Earlobe (h)	0.0005	0.0006	0.1905	0.1839	0.0005	0.0005	0.9863	0.9352
AI Jaw Angle (h)	0.0007	0.0011	0.0711	0.5084	0.0008	0.0006	0.1729	0.9172
AI Nasal wing (v)	0.0001	0.0001	0.6517	0.7547	0.0001	0.0001	0.2571	0.1951
AI Lip angle (v)	0.0001	0.0001	0.9746	0.8578	0.0001	0.0001	0.9715	0.2255
AI Earlobe (v)	0.0002	0.0003	0.3404	0.8301	0.0004	0.0002	0.0001	0.0476
AI Jaw Angle (v)	0.0003	0.0002	0.0352	0.1671	0.0002	0.0002	0.9748	0.4032
**Enface Analysis:**								
Chin Deviation	0.0165	0.0197	0.4987	0.2176	0.0051	0.0062	0.4432	0.1081
Occlusal-plane Inclination	0.0377	0.0551	0.1383	0.3677	0.0234	0.0258	0.7078	0.8059
Midline Shift	0.0280	0.0573	0.0059	0.0145	0.0195	0.0288	0.1256	0.0535

Appendix C: Acquisition of standardized 2D-Photographs of the Face

All facial photographs analyzed in the present study were acquired under strictly standardized conditions in accordance with the recommendations of the European Association for Cranio-Maxillofacial Surgery (EACMFS) (6,16). Image acquisition was performed using a full-frame digital single-lens reflex camera (Canon EOS 6D, Canon Inc., Tokyo, Japan) equipped with a fixed 100-mm focal-length lens. The camera optical axis was aligned strictly horizontally, and the camera–subject distance was fixed at 150 cm for all recordings using permanently affixed markings on the floor (Figure C.1). Subjects were positioned in a standardized upright posture with the shoulders strictly parallel to the camera sensor plane, ensuring that the body and facial midline were aligned parallel to the imaging plane. This positioning minimized rotational and translational deviations relative to the camera. For the profile photographs, the patients were rotated 90 degrees and aligned exactly parallel to the optical axis of the camera while the Frankfort horizontal plane was kept parallel to the floor.

The camera settings were identical for all subjects and predefined in accordance with the clinic's standards: ISO sensitivity was set to 200, aperture to f/18, and shutter speed to 1/125 s. All photos were taken personally by the treating medical team, using autofocus to ensure consistent sharpness for all subjects. All images were acquired in front of a uniform light blue background (RAL 5012 / Hex-color-code: #2B65EC) in accordance with the EACMFS photographic standard. The standardized photographic projections and camera settings were displayed on a reference poster in the immediate vicinity of the photographic setup. Illumination was provided by professional external flash units arranged in a standardized three-point lighting configuration. One flash unit was positioned overhead, while two lateral flash units were positioned symmetrically on the right and left sides. This configuration ensured homogeneous illumination of the facial surface and minimized shadow formation and lighting-related artifacts.

Quantitative Estimation of Depth-Related Error

Photographs follow central perspective projection, with image scale inversely proportional to object distance. Small depth differences (ΔZ) introduce relative errors $\Delta d/d \approx \Delta Z/Z$ (with Z = object distance). For the Nakamura index, all landmarks are coplanar, so depth-related distortion is theoretically zero. For dental midline evaluation, the intercanthal distance served as a reference. Assuming a maximum depth displacement of 20 mm relative to the dental structures, the resulting relative error is about 1.3% ($\Delta Z/Z = 20/1500$), corresponding to less than 1 mm linear deviation and negligible angular error. Depth-related distortion is also expected to be minimal for Brons–Mulie analysis, as landmarks lie in the facial median plane at approximately constant camera distance, analogous to frontal radiographic cephalometric analyses. Moreover, due to the use of a long fixed 100 mm focal-length lens, optical lens distortion is negligible and does not materially affect measurements. Given the long and fixed focal length, the fixed camera–subject distance, standardized settings, and strict subject alignment, depth-related errors are expected to be limited and unlikely to introduce directional distortion, remaining below clinically and statistically relevant thresholds.

Figure C.1: Professional photo studio for standardized facial imaging. Positioning was standardized using floor markings.

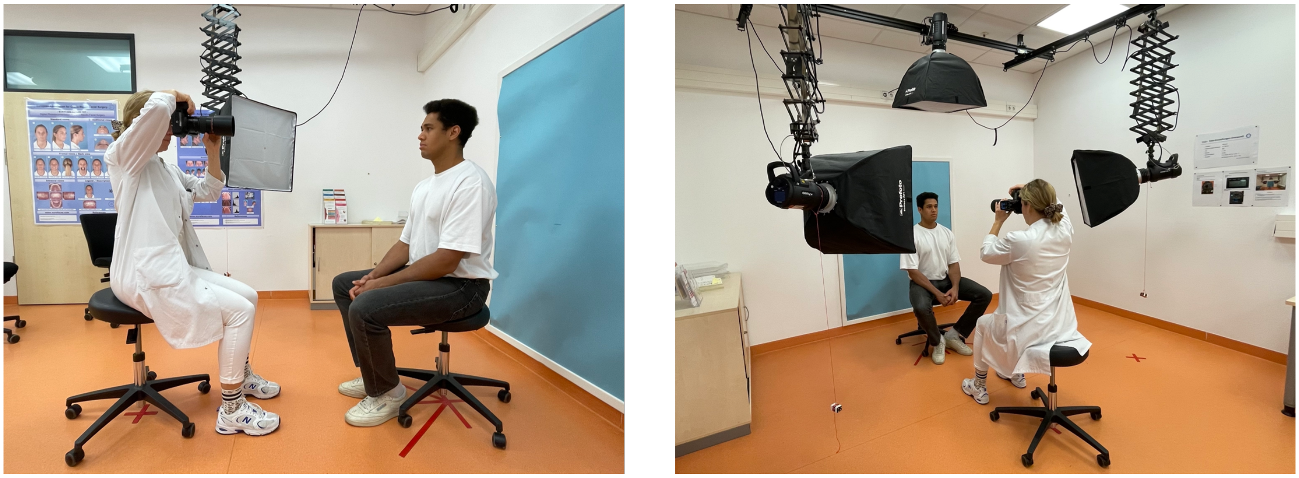

Appendix D: Inter- and Intra-Observer Reliability and Standard Error of Measurement

An analysis of inter- and intraobserver reliability was conducted in 10 patients according to the methodology described by Shrout and Fleiss (1979). For each patient, all landmarks of the Brons-Mulié Analysis, the Nakamura Asymmetry Index, and the Enface Analysis were identified using the software described in Appendix E. As the calculation of the Asymmetry Index requires bilateral facial measurements, eight parameters per side were recorded for each examination. For the Enface Analysis, in addition to the three parameters reported in the manuscript, the intercanthal distance was measured as a reference line for the comparison of pre- and postoperative images. This resulted in a total of 24 parameters per patient per examination cycle.

For the assessment of interobserver reliability, three experienced examiners independently measured all 24 parameters in the 10 patients. The intraclass correlation coefficient (ICC) was calculated according to Shrout and Fleiss (n = 10, raters = 3, two-way model, consistency type, ICC(3,1)). To assess intraobserver reliability, a single examiner measured all 24 parameters in the 10 patients at three different time points (n = 10, time points = 3, two-way model, agreement type, ICC(2,1)). The interval between measurements was 5 days. ICC values were interpreted according to Portney and Watkins (2009) (Table D.1).

Table D.1: Interpretation of ICC Values according to Portney and Watkins (2009).

ICC Value	Interpretation
<0.50	Poor reliability
0.50–0.75	Moderate reliability
0.75–0.90	Good reliability
>0.90	Excellent reliability

In the assessment of interobserver reliability, ICC values ranged from 0.361 to 0.997. Sixteen parameters demonstrated excellent reliability (ICC > 0.90), four showed good reliability (ICC 0.75–0.90), and three exhibited moderate reliability (ICC 0.50–0.75). Only one parameter, chin deviation, demonstrated poor reliability (ICC < 0.50).

For intraobserver reliability, ICC values ranged from 0.807 to 0.998. Twenty parameters demonstrated excellent reliability (ICC > 0.90), and four showed good reliability (ICC 0.75–0.90).

Overall, the majority of parameters demonstrated excellent reliability in both the inter- and intraobserver analyses. However, the determination of the parameter chin deviation showed unsatisfactory reliability in the interobserver analysis. Targeted examiner training could enhance measurement consistency for this parameter, given that intraobserver reliability was good. A complete summary of the analysis results is provided in Table D.2.

Table D.2: Inter- and intraobserver reliability for all analyzed parameters according to Shrout and Fleiss. Intraclass correlation coefficients (ICC) are reported with 95% confidence intervals and p-values. F-tests were performed for all analyses ($F_{9,18}$), $p < .01$.

	Inter-observer ICC	95% CI lower	95% CI upper	P Value	Intra-observer ICC	95% CI lower	95% CI upper	P Value
**Brons-Mulié Analysis:**								
Mandibulofacial Height	0.985	0.957	0.996	<0.01	0.993	0.979	0.998	<0.01
Upper Lip Inclination	0.859	0.651	0.959	<0.01	0.938	0.835	0.983	<0.01
Lower Lip Inclination	0.966	0.904	0.991	<0.01	0.989	0.969	0.997	<0.01
Mandibula Inclination	0.976	0.932	0.993	<0.01	0.983	0.953	0.995	<0.01
**Nakamura Index:**								
AI Nasal wing (h) right	0.916	0.777	0.976	<0.01	0.816	0.455	0.950	<0.01
AI Nasal wing (h) left	0.951	0.864	0.986	<0.01	0.954	0.869	0.987	<0.01
AI Lip angle (h) right	0.946	0.853	0.985	<0.01	0.886	0.686	0.968	<0.01
AI Lip angle (h) left	0.930	0.812	0.980	<0.01	0.941	0.844	0.983	<0.01
AI Earlobe (h) right	0.988	0.967	0.997	<0.01	0.983	0.931	0.996	<0.01
AI Earlobe (h) left	0.991	0.974	0.998	<0.01	0.988	0.966	0.997	<0.01
AI Jaw Angle (h) right	0.758	0.460	0.926	<0.01	0.902	0.476	0.978	<0.01
AI Jaw Angle (h) left	0.794	0.522	0.938	<0.01	0.902	0.720	0.973	<0.01
AI Nasal wing (v) right	0.944	0.848	0.985	<0.01	0.965	0.895	0.991	<0.01
AI Nasal wing (v) left	0.959	0.885	0.989	<0.01	0.954	0.855	0.988	<0.01
AI Lip angle (v) right	0.993	0.979	0.998	<0.01	0.994	0.979	0.998	<0.01
AI Lip angle (v) left	0.997	0.991	0.999	<0.01	0.994	0.968	0.998	<0.01
AI Earlobe (v) right	0.993	0.979	0.998	<0.01	0.998	0.994	0.999	<0.01
AI Earlobe (v) left	0.995	0.985	0.999	<0.01	0.995	0.984	0.999	<0.01
AI Jaw Angle (v) right	0.717	0.392	0.911	<0.01	0.918	0.637	0.980	<0.01
AI Jaw Angle (v) left	0.614	0.244	0.872	<0.01	0.915	0.749	0.977	<0.01
**Enface Analysis:**								
Reference line	0.974	0.927	0.993	<0.01	0.981	0.947	0.995	<0.01
Chin Deviation	0.361	-0.027	0.750	0.035	0.807	0.549	0.942	<0.01
Occlusal-plane Inclination	0.677	0.332	0.896	<0.01	0.850	0.637	0.956	<0.01
Midline Shift	0.809	0.551	0.943	<0.01	0.908	0.764	0.974	<0.01

Standard error of measurement (SEM) for each parameter was calculated using the standard deviation (SD) and the intra- and interobserver ICC obtained from the reliability analysis of the cohort of 10 patients using the following formula:

$$SEM = SD \times \sqrt{(1 - \text{ICC})}$$

Table D.3: Estimation of the standard error of measurement (SEM) based on the intra-class correlation coefficient (ICC). Linear distances were measured in pixels (px), and angular measurements in degrees (°).

	Inter-obs. ICC	Intra-obs. ICC	Inter-obs. SEM	Intra-obs. SEM	Unit
**Brons-Mulié Analysis:**					
Mandibulofacial Height	0.985	0.993	1.50	1.02	px
Upper Lip Inclination	0.859	0.938	3.11	1.85	°
Lower Lip Inclination	0.966	0.989	1.85	1.00	°
Mandibula Inclination	0.976	0.983	1.70	1.36	°
**Nakamura Index:**					
AI Nasal wing (h) right	0.916	0.816	6.96	8.65	px
AI Nasal wing (h) left	0.951	0.954	7.33	8.00	px
AI Lip angle (h) right	0.946	0.886	10.55	15.54	px
AI Lip angle (h) left	0.930	0.941	11.58	11.37	px
AI Earlobe (h) right	0.988	0.983	8.62	10.31	px
AI Earlobe (h) left	0.991	0.988	7.27	8.16	px
AI Jaw Angle (h) right	0.758	0.902	36.83	23.75	px
AI Jaw Angle (h) left	0.794	0.902	35.52	23.78	px
AI Nasal wing (v) right	0.944	0.965	13.77	10.60	px
AI Nasal wing (v) left	0.959	0.954	11.57	12.25	px
AI Lip angle (v) right	0.993	0.994	7.78	7.01	px
AI Lip angle (v) left	0.997	0.994	4.63	6.61	px
AI Earlobe (v) right	0.993	0.998	12.68	6.62	px
AI Earlobe (v) left	0.995	0.995	9.91	9.37	px
AI Jaw Angle (v) right	0.717	0.918	68.03	35.73	px
AI Jaw Angle (v) left	0.614	0.915	68.56	34.30	px
**Enface Analysis:**					
Reference line	0.974	0.983	9.36	7.82	px
Chin Deviation	0.361	0.796	0.79	0.34	°
Occlusal-plane Inclination	0.677	0.843	0.64	0.38	°
Midline Shift	0.809	0.914	11.97	9.45	px

Appendix E: Digital Implementation of Analysis Methods – Software Pipeline

All three analysis methods—the Brons-Mulié analysis, the Nakamura asymmetry index, and the enface analysis—were performed digitally. Although, in principle, the analyses can be carried out on any standard monitor, a 27-inch display was used to ensure maximum precision.

The Brons-Mulié analysis was implemented in Java using a custom-developed application. Analyses were performed using the Oracle Java Development Kit (JDK; Oracle Corporation, Austin, TX, USA), with the respective current stable release at the time of analysis. On the profile image, the examiner manually identifies the anatomical landmarks required for the analysis. The corresponding lines, angles, and reference ranges are then calculated automatically by the software in accordance with the method described by Brons and Mulié (Figure F.1). The resulting values were subsequently transferred manually or via copy-and-paste to Microsoft Excel (Microsoft Corporation, Redmond, WA, USA) for further analysis.

For assessment of the Nakamura asymmetry index and the enface analysis, the software ImageJ was used in its respective current version (ImageJ; National Institutes of Health, Bethesda, MD, USA). ImageJ allows the placement of points and lines on digital images and their automated evaluation using JavaScript-based macros (Figure F.1). For both analyses, dedicated scripts were developed to automatically perform the required length and angle measurements and to display the results in a separate output window for data extraction. The resulting data could then be easily transferred to Microsoft Excel via copy-and-paste for further statistical evaluation.

Figure F.1: Screenshots of the custom-developed Java application (right) and the ImageJ software (left).

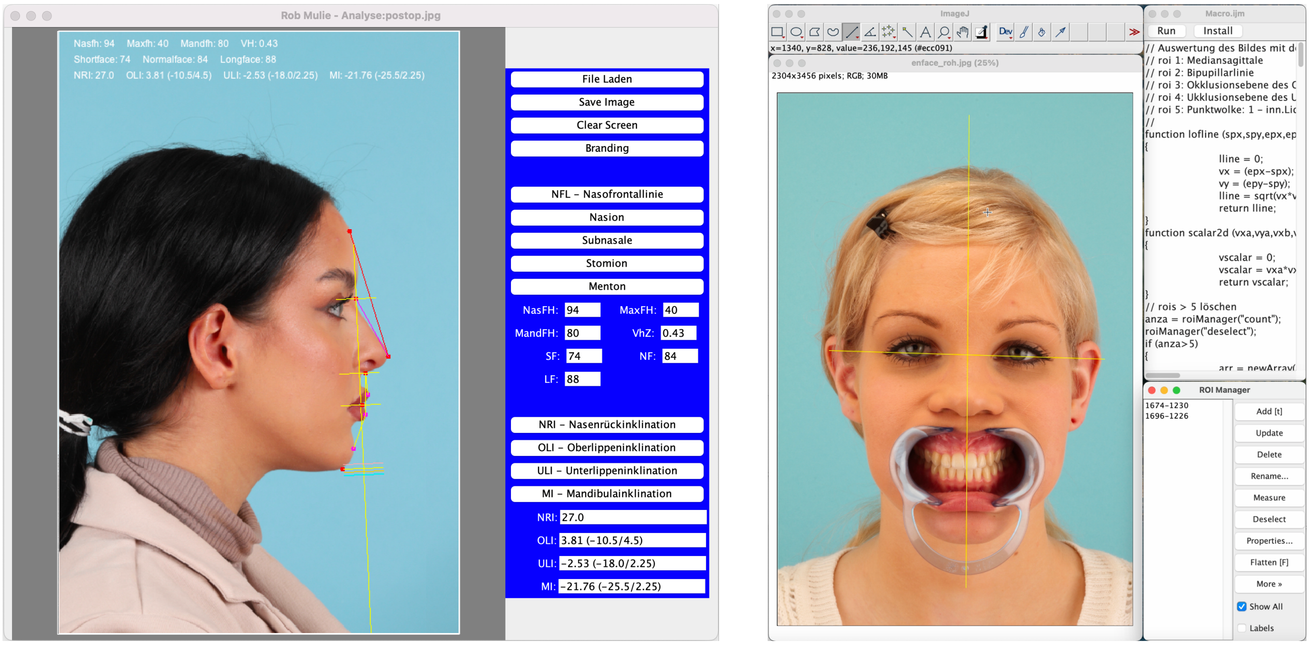

Supplement: Supplementary file 1 [file Datasheet1.pdf]
