# Supplementary figures and images for: Quality assurance in orthognathic surgery using Brons-Mulié's soft tissue analysis, Nakamura's asymmetry index and a simple enface analysis
Source: Front Oral Health. 2026 Mar 26;7:1748425. doi: 10.3389/froh.2026.1748425 (PMC13062170; doi:10.3389/froh.2026.1748425)

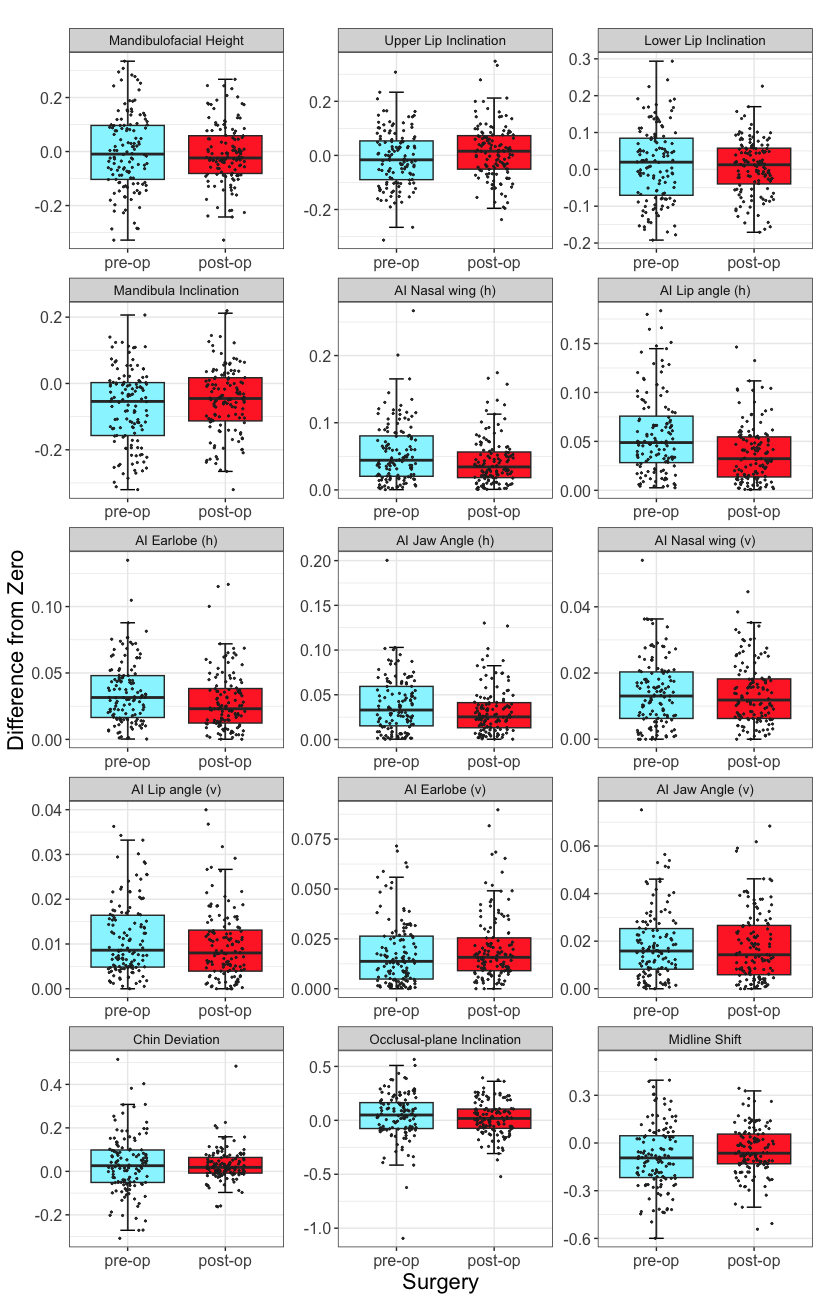

Supplement: Supplementary file 2 [file Image1.jpeg]

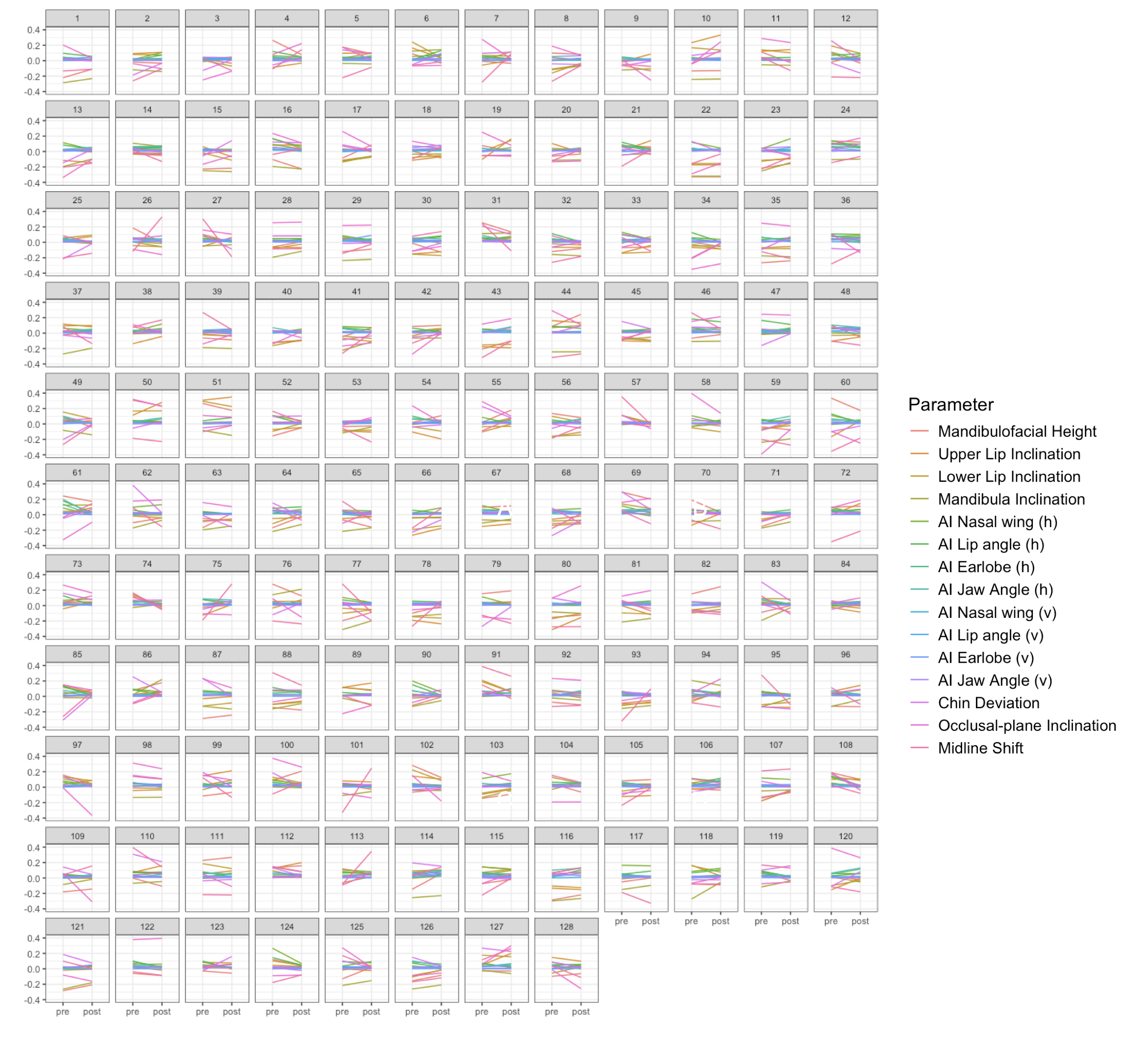

Supplement: Supplementary file 3 [file Image2.jpeg]
